# Segmentation of human functional tissue units in support of a Human Reference Atlas

**DOI:** 10.1038/s42003-023-04848-5

**Published:** 2023-07-19

**Authors:** Yashvardhan Jain, Leah L. Godwin, Yingnan Ju, Naveksha Sood, Ellen M. Quardokus, Andreas Bueckle, Teri Longacre, Aaron Horning, Yiing Lin, Edward D. Esplin, John W. Hickey, Michael P. Snyder, Nathan Heath Patterson, Jeffrey M. Spraggins, Katy Börner

**Affiliations:** 1 Department of Intelligent Systems Engineering, Luddy School of Informatics, Computing, and Engineering, Indiana University, Bloomington, IN 47408 USA; 2 Department of Pathology, Stanford University School of Medicine, Stanford, CA 94305 USA; 3 Thermo Fisher Scientific, South San Francisco, CA 94080 USA; 4 Department of Surgery, Washington University School of Medicine, St. Louis, MO 63110 USA; 5 Department of Genetics, Stanford University School of Medicine, Stanford, CA 94305 USA; 6 Department of Microbiology & Immunology, Stanford University School of Medicine, Stanford, CA 94305 USA; 7 Mass Spectrometry Research Center, Vanderbilt University, Nashville, TN 37232 USA; 8 Department of Cell and Developmental Biology, Vanderbilt University, Nashville, TN 37232 USA

**Keywords:** Data processing, Machine learning

## Abstract

The Human BioMolecular Atlas Program (HuBMAP) aims to compile a Human Reference Atlas (HRA) for the healthy adult body at the cellular level. Functional tissue units (FTUs), relevant for HRA construction, are of pathobiological significance. Manual segmentation of FTUs does not scale; highly accurate and performant, open-source machine-learning algorithms are needed. We designed and hosted a Kaggle competition that focused on development of such algorithms and 1200 teams from 60 countries participated. We present the competition outcomes and an expanded analysis of the winning algorithms on additional kidney and colon tissue data, and conduct a pilot study to understand spatial location and density of FTUs across the kidney. The top algorithm from the competition, Tom, outperforms other algorithms in the expanded study, while using fewer computational resources. Tom was added to the HuBMAP infrastructure to run kidney FTU segmentation at scale—showcasing the value of Kaggle competitions for advancing research.

## Abstract (web summary)

Results from a Kaggle competition and expanded analysis of the winning algorithms are presented for segmentation of functional tissue units as part of the Human BioMolecular Atlas Program (HuBMAP).

## Introduction

The Human BioMolecular Atlas Program (HuBMAP) aims to create an open, computable Human Reference Atlas (HRA) at the cellular level^1^. The envisioned HRA^2^ will make it possible to register and explore human tissue data across scales—from the whole-body macro-anatomy level to the single-cell level. Medically relevant functional tissue units (FTUs) are seen as important for bridging the meter-level scale of the whole body to the micrometer scale of single cells. De Bono et al.^3^ defined FTUs in support of tissue modeling as “a three-dimensional block of cells centered around a capillary where each cell is within diffusion distance from any other cell within the same block.” Here, we are interested in capturing major anatomical structures and cell types for key FTUs to construct the HRA; thus, we define FTUs as the smallest tissue organization that performs a unique physiologic function and is replicated multiple times in a whole organ. Note that the HRA keeps track of structural biology data and 3D spatial data—i.e., for each major FTU there exists information on its anatomical structures, cell types, and biomarkers in the ASCT + B tables and a 2D illustration that characterizes the general number, size, shape, and spatial location of cell types to each other.

The value of FTUs is acknowledged by the scientific and medical communities, yet limited data exists about human diversity (e.g., the number and size distribution of FTUs for a single organ and across individuals with different age, sex, body mass index [BMI]). A key reason for this knowledge gap is the fact that manual segmentation of FTUs is time-consuming, expensive, and does not scale to the data now available. For example, there are over 1 million glomeruli in an average human kidney^4^, but a trained pathologist needs ca. 50 min of time to segment 200 FTUs (~15 s per FTU)—a value computed by averaging the time spent by four experts segmenting about 12,900 FTUs across five organs for a different Kaggle competition^5^. FTU detection algorithms exist^6–18^ and approaches range from simple thresholding to deep-learning methods. Existing methods achieved varying levels of performance (see Supplementary Tables 1 and 2 and performance metric definitions in “Methods”) and face challenges when applied to human data (e.g., training on murine glomerulus data generated false positives when applied to the much larger glomeruli in human data^14^). There has been much high-quality work done in the field of glomerular detection and segmentation through deep-learning methods, such as approaches that perform better for model species than humans^15^, focus on detecting disease states^16,17^, or focus on rapid FTU detection^18^. Recent work by Lutnick et al.^19^ makes a strong case for using deep-learning algorithms for segmentation of glomeruli in histology images by using a Deeplab-v2 architecture on a dataset containing both human (healthy and diseased) and murine samples. Rapid progress that focuses on the healthy human adult is desirable as a robust and highly performant FTU segmentation model would make it possible to compute size, shape, and variability in number, size, and location of FTUs within tissue samples. Results are needed to characterize human diversity, providing critical information for the construction of a spatially accurate and semantically explicit model of the human body that could then be compared to disease states. HuBMAP holds at its core the goal to provide FAIR^20^ data and open-source tools for the construction of said HRA to ensure full transparency and reproducibility. Hence, this study focuses on an evaluation of non-proprietary code using various state-of-the-art deep-learning segmentation architectures.

As part of the Human BioMolecular Atlas Program (HuBMAP), we designed and hosted a Kaggle competition that focussed on developing open-source machine-learning solutions for segmentation of non-sclerotic renal glomeruli in PAS-stained histological images of human kidney tissue. 1200 teams from 60 countries contributed code and expertise and submitted 34,699 solutions. We downloaded the winning solutions (see “Competition design” in Methods), reproduced performance results, and compared algorithm runtime. Next, we conducted a generalizability study on additional FTU types and datasets. Finally, we productized the top algorithm from the competition and added it to the HuBMAP data analysis infrastructure (https://hubmapconsortium.org/infrastructure/) where it is now being run at scale on human kidney tissue data provided by various Tissue Mapping Centers (TMCs). FTU segmentation, in conjunction with the spatial location of the tissue within the kidney, can be used to compute diversity and distribution of FTUs in kidneys across the human population and will aid the creation of the HRA, driven by critical real-world data. In the future, we plan to extend this work to other organs in the human body.

**Fig. 1:**
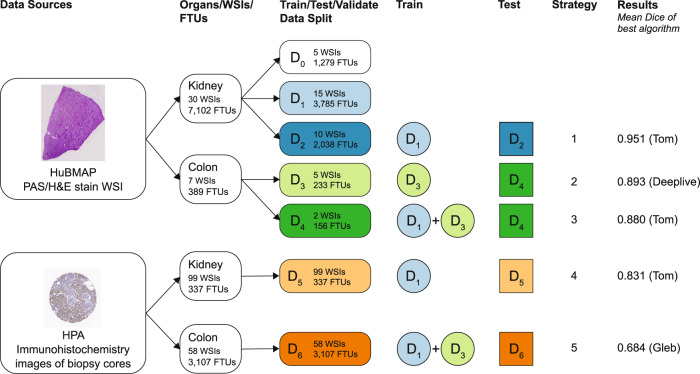
Overview of algorithm comparison setup using HuBMAP and HPA data. Five different training and test strategies were implemented. Strategy 1: In the “HuBMAP - Hacking the Kidney” Kaggle competition, the HuBMAP kidney dataset was split into 15 WSIs for training, 10 for test, and 5 for validation. Strategy 2: To showcase generalizability, winning algorithms are trained on 5 HuBMAP colon WSIs and then tested on 2 colon WSIs. Strategy 3: Transfer learning uses 15 kidney WSIs (pretrained model from Strategy 1 is used as initial weights), 5 colon WSIs to train the model, and 2 colon WSIs to test—all data is from HuBMAP. Strategy 4 and 5 use HPA data for kidney and colon data, respectively, to test inference-time generalizability across stains without further training; Strategy 4 uses the models trained in Strategy 1 and tests on 99 HPA kidney WSIs; Strategy 5 uses the models trained in Strategy 3 and tests on 58 HPA colon WSIs.

This paper is organized as follows: (1) we reproduce results (using same hyperparameter values and data split as used in the competition) from the “HuBMAP – Hacking the Kidney” Kaggle competition^21^ for the five winning algorithms on the renal glomerulus dataset; (2) we apply all five algorithms to the colonic crypts dataset (with and without transfer learning) to determine their generalizability to other FTU types; (3) we test inference of all models trained on both renal glomerulus and colonic crypts datasets (using transfer learning) on a kidney and colon immunohistochemistry dataset from the Human Protein Atlas^22,23^ (HPA) without any additional training on this dataset to compare cross-dataset inference generalizability of learned features across different tissue staining methods (see “Methods” section for details and Fig. 1 for on overview of all five strategies); (4) we present a pilot study that uses the segmentation data to characterize the number of FTUs per unit area in dependence on location in the human body as well as donor sex, age, and BMI; (5) we discuss how FTU segmentation advances the construction of the HRA. We have made all data (including ground truth segmentations) and the validated code (with trained models) freely accessible at https://github.com/cns-iu/ccf-research-kaggle-2021.

## Results

### Data preparation

**Fig. 2:**
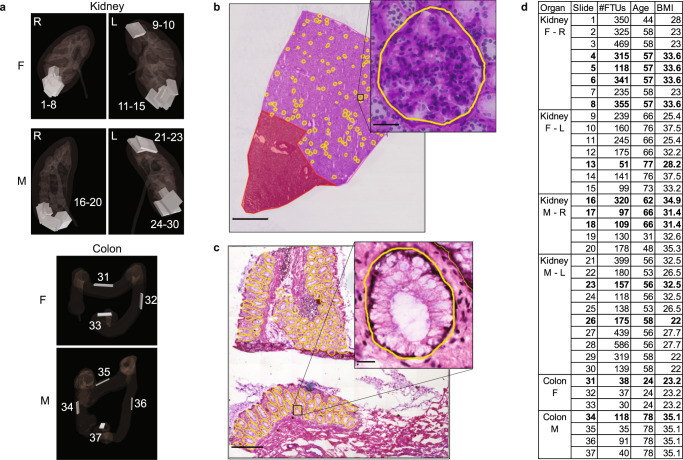
FTU datasets. **a** The 30 kidney and 7 colon tissue datasets were registered into the corresponding male/female, left/right HuBMAP 3D reference organs for kidney and colon to capture the size, position, and rotation of tissue blocks. **b** Sample kidney WSI (scale bar: 2 mm) with zoom into one glomerulus annotation (scale bar: 50 µm). **c** Sample colon WSI (scale bar: 500 µm) with zoom into a single crypt annotation (scale bar: 20 µm). **d** Metadata for 37 WSIs sorted top-down by vertical location within the reference organs; test datasets are given in bold.

For the “HuBMAP – Hacking the Kidney” Kaggle competition, a dataset was compiled comprising 30 Periodic acid-Schiff (PAS) stain whole slide images (WSIs) with 7102 segmented renal glomeruli (see Supplementary Table 3 and “Methods”). To determine if algorithms generalize to other FTU types, a second dataset was compiled comprising seven colon hematoxylin and eosin (H&E) stain WSIs with 395 segmented colonic crypts (see Supplementary Table 4 and “Methods”). This renal glomeruli and colonic crypt dataset is collectively referred to as “the HuBMAP data” in the paper. Figure 2a shows the tissue extraction sites for the 30 kidney and 7 colon WSI datasets (explore three-dimensional reference organs at https://cns-iu.github.io/ccf-research-kaggle-2021). Exemplary renal glomerulus and colonic crypt segmentations are given in Fig. 2b. Figure 2d lists basic information (sex, age, BMI, number of FTUs) for all 37 WSIs, sorted by their vertical (y-axis) spatial location in the reference organ, using the mass point of the tissue block from which they were extracted (top-most block is on top); see “Methods”.

The dataset for the Kaggle competition, which consisted of 30 WSIs containing renal glomeruli, was split into three sets: a training set containing 15 WSIs with 3785 FTUs, a validation set (public test set) containing 5 WSIs with 1279 FTUs, and a private test set containing 10 WSIs with 2038 FTUs. The training set and the validation set were available to competition participants. The private test dataset was used for scoring algorithm performance (see competition design in “Methods”). Analogously, the colon dataset was split into a training set containing 5 WSIs and a test set containing 2 WSIs containing 233 and 156 colonic crypts, respectively. All test datasets are rendered in bold in Fig. 2c.

To understand the cross-dataset inference generalizability of learned features across a different tissue staining methods, we compiled a third dataset from the HPA^22,23^ consisting of 99 kidney and 58 colon immunohistochemistry (IHC) WSIs. The 337 renal glomeruli in kidney WSIs and the 3107 colonic crypts in colon WSIs were manually segmented by experts. This dataset is referred to as “the HPA data” in the paper.

### Algorithm comparison

#### Winning strategies

The five winning algorithms from the “HuBMAP - Hacking the Kidney” Kaggle Competition are from teams named Tom, Gleb, Whats goin on, DeepLive.exe, and Deepflash2. All five algorithms use a U-Net-style architecture with different classification models as encoders for feature extraction.

##### Tom

The Tom model consists of a single U-Net-style architecture with SeResNext101^24,25^ (a ResNext model with a Squeeze-and-Excitation block) as the encoder, a Convolutional Block Attention Module (CBAM)^26^, hypercolumns, and deep supervision. The loss function used is defined as a combination of Binary Cross-entropy loss^27^ and Lovász Hinge loss^28^, and the optimizer used is SGD (stochastic gradient descent)^29^. In addition to the provided training data, the model is trained on additional data hosted on Mendeley^30^ and the HuBMAP Data Portal^31^ (2 WSIs).

##### Gleb

The Gleb model uses an ensemble of four 4-fold models: Three U-Net-style models with different encoders (namely, RegnetY-16, RegnetX-32, and RegnetY-16) and an scSE^24^ attention decoder, and 1 U-Net++ style model with RegnetY-16 as the encoder and an scSE attention decoder. All models used belong to the Regnet^32^ family of models. It uses data parallelism to utilize all available GPUs on the system for model training. The loss function used is the Dice coefficient loss^33^ and the optimizer used is AdamW^34^.

##### Whats goin on

This model is an ensemble of two U-Net-style 5-fold models with Resnet50 and Resnet101^35^ (both using the 32x4d version) as encoders. In addition, a Feature Pyramid Network (FPN)^36^ is added to provide skip connections between upscaling blocks of the decoder, atrous spatial pyramid pooling (ASPP)^37^ is added to enlarge receptive fields, and pixel shuffle^38^ is added instead of transposed convolution to avoid artifacts. The loss function used is the binary cross-entropy loss, with gradient norm clipping at 1, and the optimizer used is Adam^39^.

##### DeepLive.exe

A single U-Net-style model with EfficientNet-B1^40^ as encoder is used. In addition to the provided training data, the model is trained on additional data hosted on Mendeley^30^ (31 WSIs), Zenodo^41^ (20 WSIs), and the HuBMAP Data Portal^42^ (2 WSIs). The additional data is classified into two classes: healthy and unhealthy glomeruli. The model employs a dynamic sampling approach whereby it samples tiles of size 512 × 512 pixels (at a resolution downscale factor of 2) and 768 × 768 pixels (at a resolution downscale factor of 3). The tiles are sampled from regions having visible glomeruli in them based on segmentations, instead of sampling randomly. Model training uses the cross-entropy loss and the Adam^39^ optimizer. This approach tries to reframe the problem as a healthy/unhealthy glomerulus classification problem along with a segmentation problem. This setup enables the model to learn to classify the unhealthy glomeruli as glomeruli and then decide whether the particular instance is healthy enough.

##### Deepflash2

The model used is a simple U-Net-style architecture with an EfficientNet-B2^40^ as the encoder. Input data is converted and stored in a *.zarr file format for efficient loading on runtime. The model collectively employs two sampling approaches: (1) Sampling tiles that contain all glomeruli (to ensure that each glomerulus is seen at least once during each epoch of training). (2) Sampling random tiles based on region (cortex, medulla, background) probabilities (to give more weight to the cortex region during training since glomeruli are mainly found in the cortex). The region sampling probabilities were chosen based on expert knowledge and experiments: 0.7 for cortex, 0.2 for medulla, and 0.1 for background. Model training uses a weighted sum of Dice^43^ and cross-entropy loss^44^ (where both losses have equal weight), Ranger^45^ optimizer (a combination of RAdam^46^ and LookAhead optimizer^47^). The best model ensemble for the final score consists of three models trained on different zoom scales (i.e., 2×, 3×, 4×). This team also had access to a biomedical expert who was part of the team.

In sum, to achieve high performance, algorithms use an ensemble of multiple machine learning models (Gleb, Whats goin on), specialized sampling strategies (DeepLive.exe, Deepflash2), addition of auxiliary tasks (healthy/unhealthy glomeruli classification in DeepLive.exe), and/or specialized machine learning techniques such as deep supervision, hypercolumns, attention modules, pixel shuffle etc. Since most data augmentation techniques used by all algorithms are similar (see further algorithm details in “Methods”), it is these specialized strategies that give each model its performance boost over the solutions by the other nearly 1200 teams. Tom, the performance prize winner as well as the best-performing model in our transfer learning and generalizability study, showcases single model architectures that employ specialized machine learning techniques (Convolutional Block Attention Module, Hypercolumns, Deep supervision) can be as competitive and efficient, if not better, than multiple model ensembles (a popular strategy used in Kaggle competitions to improve leaderboard score). This is important since single model architectures can drastically reduce computation time and resource usage, and are easier to debug, which is essential for productizing algorithms. While Deeplive.exe did perform as well as Tom in some cases (see Performance and Generalizability), the latter is superior due to much lower resource requirements and is therefore selected for integration and usage in the HuBMAP data analysis infrastructure.

#### Performance

Performance values for each of the five algorithms without using the watershed segmentation are provided for comparison in Supplementary Table 6. Supplementary Table 7 lists Dice coefficients, false negatives (FN), true positives (TP), and false positives (FP) of winning algorithms for individual WSIs in all three predicted datasets (10 kidney WSIs using Strategy 1; 2 colon WSIs using Strategy 2; 2 colon WSIs using Strategy 3, see Fig. 1 for an explanation of all strategies). Threshold used for calculations is 0.5. For watershed, a minimum distance of 30 for kidney and 60 for colon data is used. Interactive data visualizations can be explored at https://cns-iu.github.io/ccf-research-kaggle-2021.

**Fig. 3:**
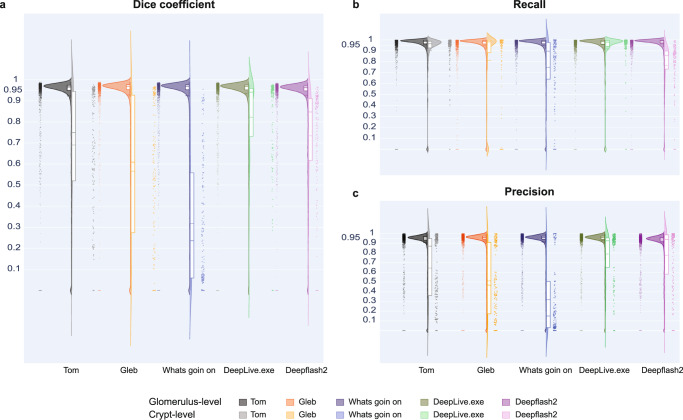
Algorithm performance results on HuBMAP data. Violin plots show performance for kidney on the left (one dot per 2038 glomeruli) and transfer-learning performance for colon data (one dot for each of the 160 crypts) on the right. **a** Dice coefficient. **b** Recall. **c** Precision. Mean Dice values per WSI for all algorithms are provided in Supplementary Table 7. Interactive versions of these graphs are at https://cns-iu.github.io/ccf-research-ftu/hubmap_violin.html.

Performance results with watershed are shown in Fig. 3 using violin plots for three metrics: Dice, precision, and recall, see details in the “Methods” section. For each metric, we show distribution for the ten kidney WSIs with 2038 glomeruli on the left and the distribution for the two colon WSIs with 160 crypts (pretrained on renal glomeruli data) on the right. Performance on kidney vs. colon data can be easily compared. As expected, due to the smaller size of the colon data, all five algorithms have a higher Dice coefficient for kidney data than for colon data. Tom has the highest mean Dice score of 0.951 on the kidney data. It also has the highest precision value of 0.979, with 95 false negatives and 41 false positives out of 2038 glomeruli. As for recall, DeepLive.exe has the highest value of 0.969, with 63 false negatives and 70 false positives. Tom has the highest mean Dice score of 0.880, followed by DeepLive.exe with a value of 0.877, for colon data pretrained on kidney data. As for recall and precision, DeepLive.exe has the highest value with 0.84 and 0.92, respectively, with 26 false negatives and 11 false positives out of 160 crypts. The crypt segmentation solution for Tom in comparison with ground truth for colon data can be explored at https://cns-iu.github.io/ccf-research-kaggle-2021.

Runtime performance was recorded for the training phase on kidney data, colon data without pretraining on kidney data, and colon data with pretraining on kidney data (see Table 1). We also report runtime for the two prediction (inference) tasks: kidney data (10 WSIs) and colon data pretrained on kidney data (2 WSIs, pretrained on 15 kidney WSIs followed by training on five colon WSIs); see “Methods” section for details.

**Table 1:** Approximate runtime performance for training and prediction runs for all five algorithms on HuBMAP data.

Model	Training on kidney data (*n* = 15)	Training on colon data (no pretraining on kidney data) (*n* = 5)	Training on colon data (pretrained on kidney data) (*n* = 5)	Inference on kidney data (*n* = 10)	Inference on colon data (*n* = 2)
Tom	23 h	2 h	2.5 h	1.3 h	1.3 min
Gleb	8 h	4 h	4 h	0.4 hs	2 min
Whats goin on	24 h	0.5 h	0.5 h	0.75 h	1.75 min
DeepLive.exe	51 h	45 h	45 h	1 h	2 min
Deepflash2	8 h	1.6 h	1 h	0.22 h	0.35 min

All models were run on Indiana University’s Carbonate large-memory compute cluster, using the GPU partition which consists of 24 Apollo 6500 GPU-accelerated nodes where each node is equipped with two Intel 6248 2.5 GHz 20-core CPUs. We used a single node with 30 GB of RAM and 4 Nvidia V100-PCIE-32GB GPUs for all models except DeepLive.exe, which was run on a single node with 300 GB of RAM and 2 GPUs because it required a lot more memory for model training. All models utilize only a single GPU for training except Gleb, which uses data parallelism to distribute training across all 4 GPUs.

As can be seen, training requires longer runtimes (8–51 h for kidney and 0.5–45 h for colon) while prediction runs are much faster (0.22–1.3 h for kidney and 0.35–2 min for colon). Total algorithm runtime (training on kidney, then colon plus inference on colon) is lowest for Deepflash2 (9 h), followed by Gleb (12 h), Whats goin on (24.5 h), and Tom (25.5 h). Deepflash2 (0.22 h) and Gleb (0.4 h) are the fastest in kidney prediction.

The transfer learning strategy (Strategy 3) used in this paper shows little to no impact of pretraining on HuBMAP kidney data on predictions on HuBMAP colon data (see Supplementary Table 7). We hypothesize this may be the result of the extremely limited number of samples in the HuBMAP colon data and acknowledge this as a limitation of our work (see “Limitations” section).

#### Generalizability

HuBMAP provides an open Data Portal that is used by many different research teams to ingest many different types of data, including spatially explicit image-based tissue samples collected using different tissue staining methodologies (PAS, H&E, IHC etc.). Since the data used for HRA construction depends on the data within the HuBMAP Data Portal, there is a need to build segmentation models that can take advantage of these different tissue staining methods, especially since some types may be more prevalent than others. In cases where ground truth segmentations for data using a particular stain are not available for training a model, generating good enough pseudo-segmentations from models trained on a different stain which are then further improved manually by SMEs would lead to a reduction in resource usage (time, money) for training data curation.

To determine the cross-dataset inference generalizability of learned features across different modalities and staining methods, all five algorithms were used to run inference (i.e., predict FTU segmentations) on an immunohistochemistry dataset from the HPA (see data details in “Datasets” in Methods section). We used the models trained on HuBMAP kidney and colon data and predicted renal glomeruli and colonic crypts in the HPA data comprising 99 kidney and 58 colon WSIs. In general, the five models perform much better on the kidney data than on the colon data. We hypothesize that this might be due to the much higher quantity of HuBMAP kidney data than HuBMAP colon data used for model training.

Performance results for all algorithms on kidney HPA data are tabulated in Supplementary Table 8. This table lists Dice coefficients, false negatives (FN), true positives (TP), and false positives (FP) of winning algorithms for individual WSIs in kidney HPA data (using Strategy 4, see Fig. 1). Threshold used for calculations is 0.5. For watershed, a minimum distance of 80 is used. Overall, Tom has the highest mean Dice score of 0.83, followed by DeepLive (0.74) and Gleb (0.73). In terms of precision, Gleb performs best with 0.94, with 13 false positives, 217 true positives, and 71 false negatives out of a total of 337 glomeruli. In terms of recall, Tom performs best with 0.78, with 58 false positives, 263 true positives, and 73 false negatives. Deepflash2 suffers a drastic drop in performance on this dataset, getting a mean Dice score of 0.07, precision of 0.01, and recall of 0.18.

**Fig. 4:**
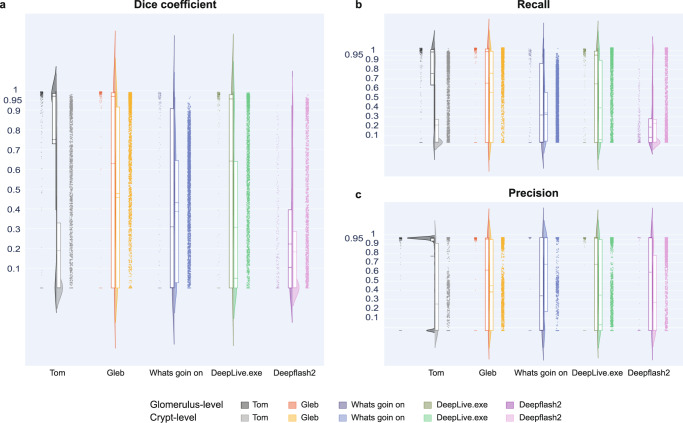
Algorithm performance results on HPA data. Violin plots show performance for kidney on the left (one dot per 337 glomeruli) and colon data (one dot for each of the 3107 crypts) on the right. **a** Dice coefficient. **b** Recall. **c** Precision. Mean Dice values for all algorithms are provided in Supplementary Tables 7 and 8. Interactive versions of these graphs are at https://cns-iu.github.io/ccf-research-ftu/hpa_violin.html.

For HPA colon data, performance results for all algorithms are listed in Supplementary Table 9. This table lists Dice coefficients, false negatives (FN), true positives (TP), and false positives (FP) of winning algorithms for individual WSIs in colon HPA data (using Strategy 5, see Fig. 1). Threshold used for calculations is 0.5. For watershed, a minimum distance of 80 is used. Overall, Gleb has the highest mean Dice score of 0.68, followed by DeepLive.exe (0.48) and Whats goin on (0.44). In terms of precision, Gleb performs best with 0.88, with 182 false positives, 1411 true positives, and 1690 false negatives out of a total of 3107 crypts. In terms of recall, as well, Gleb performs best with a score of 0.45. Tom’s performance drops drastically for this dataset, getting a mean Dice score of 0.20, precision of 0.51, and recall of 0.18. Performance results are shown in Fig. 4 using violin plots for three metrics: Dice, precision, and recall (see details in the “Methods” section and interactive data visualization at https://cns-iu.github.io/ccf-research-kaggle-2021).

These results show that inference-time feature transfer is possible for a particular FTU across different tissue staining methods, which is an important result for HuBMAP and construction of the HRA. Using additional training of the models on this data, the performance can be further improved. In addition, better stain normalization and data augmentation techniques are needed to improve performance on this task, which is the focus of the second Kaggle competition entitled “HuBMAP + HPA - Hacking the Human Body” (https://www.kaggle.com/competitions/hubmap-organ-segmentation) concluded in 2022.

### Pilot study on characterizing human diversity

Understanding the spatial location and density of FTUs across organs is critically important for advancing the construction of the HRA^2^. A robust and highly performant FTU segmentation algorithm makes it possible to compute the size, shape, variability in number, and location of FTUs within tissue samples. This information can then be used to characterize human diversity, to decide on what tissue data should be collected to improve the coverage and quality of the HRA, and for quality control (e.g., FTU size and density that is vastly different from normal might indicate disease, problems with data preprocessing, or segmentation algorithms).

In this study, we use 30 HuBMAP kidney WSI datasets from 16 donors. A total of 7102 glomeruli segmentations were identified for those 30 WSI. We are interested in observing how the number of FTUs per square millimeter varies with location-of-tissue in the human kidney reference, while accounting for sex, age, BMI, etc. The answer to this question will help guide future tissue sampling strategies. Supplementary Fig. 1 shows the relationship of tissue location and age on the number of detected glomeruli within the 16 donors. Blocks that have the same age are from the same donor. Slides are numbered by their vertical location in the 3D kidney reference organ, see also Fig. 2a. As can be seen, out of the 8 females, one has 4 tissue blocks (in y-sequence, top-down: 5, 6, 8, 4), one has 3 tissue blocks (3, 2, 7), and two have 2 tissue blocks (9, 11; 10, 14). For the 8 males, two have 3 tissue blocks (in y-sequence: 21, 23, 24; 29, 30, 26) and three have 2 tissue blocks (25, 22; 28, 27; 18, 17). In some cases, the number of glomeruli per mm^2^ decreases for females and increases for males (except for HBM 322:KQBK.747) going from top to bottom of the kidney. However, this study is provided as a pilot and more data is needed to arrive at statistically significant results (see “Limitations”).

### Limitations

Known limitations for characterizing human diversity include data sparsity and opportunistic tissue data sampling. Due to the small number of donors, we acknowledge no statistical claims can be made regarding human diversity but we provide the results of a pilot study. Going forward, as more diverse data is incorporated into the HuBMAP data portal, this pilot study will be expanded across datasets to compute statistically significant results that can guide the systematic construction of the HRA. In addition, we plan to use HRA data to guide decisions on what tissue data should be collected in terms of donor characteristics (e.g., sex, age, BMI), spatial location, and assay type (bulk or spatially explicit) to improve the coverage and quality of the HRA.

While the data used in and published with this paper is a comprehensive fully segmented FTU dataset, it is still comparatively small—particularly the HuBMAP colon dataset. We integrated FTU segmentation code into the HuBMAP portal so it can be run over all future kidney PAS stain WSI data and to increase transfer-learning performance.

The watershed algorithm used to separate neighboring FTUs requires some manual tuning for each dataset. In addition, the watershed algorithm fails to separate all FTUs in an image in case of more complex structures, such as colonic crypts which may vary in size and shape across an image. To overcome this, instance segmentation^48^, which detects and segments each instance of all objects, or panoptic segmentation^49^, which combines semantic and instance segmentation, can be used instead of semantic segmentation (which assigns a class label to each pixel).

Since HuBMAP focuses on building a reference atlas of a healthy human, we aimed to build a dataset containing segmentations for healthy glomeruli while excluding sclerotic glomeruli. Since the level of injury is on a continuous scale, some segmented glomeruli are not perfectly healthy and hence, the HuBMAP kidney dataset contains some noisy labels.

The limited amount of HuBMAP colon data used for transfer learning (Strategy 3 in Fig. 1) study makes it difficult to analyze the impact of pretraining on kidney data, and should be further explored in future work with a larger dataset.

## Discussion

There is a need for open-source code that efficiently and accurately segments healthy FTUs across organs for tissue data providers within HuBMAP and the broader biomedical community. This paper compares the winning algorithms from the “HuBMAP - Hacking the Kidney” Kaggle competition which concluded in 2021 and identifies Tom and Gleb algorithms as the most accurate, generalizable, and runtime-efficient algorithms.

To our knowledge, this is the first time that scientific evidence is provided for the value of community-driven competitions such as Kaggle competitions to develop state-of-the-art algorithms. The 1200 teams that participated in the competition performed many iterations of experimentation in an extremely collaborative manner, sharing resources, ideas, and code to arrive at highly efficient solutions. The participants included academic researchers, data scientists, and machine-learning engineers from all over the world. Given the success of this first competition, we opened a new Kaggle competition in 2022 titled “Hacking the Human Body” in collaboration with the HPA team that aims to advance more generalized FTU segmentation algorithms for five organs that have FTUs of markedly different shapes and sizes and across WSIs with different stains, resolutions, and tissue thicknesses^21^.

All code used in this study has been documented and made available freely for anyone to use. We added the winning algorithm for production usage in the HuBMAP Data Portal^42^ and made it available as part of the HRA ecosystem. Soon, kidney datasets that were spatially registered using the HuBMAP registration user interface^31^ and that have anatomical structures in which FTUs are known to exist will automatically be segmented.

Going forward, 3D data of FTUs will be used to identify the number, size, and shape of FTUs in support of machine learning and single-cell simulation of the structure and function of FTUs in human tissue. Resulting data will be used to increase our collective understanding of (and variability in) the size, number, and location of FTUs in relation to donor sex, age, ethnicity, and BMI. This data and work is also critical for top-down (segmenting out larger known structures) and bottom-up (single-cell) data integration and analysis that are needed for constructing an accurate and comprehensive HRA.

## Methods

### Datasets

#### HuBMAP and HPA renal glomeruli data

Renal glomeruli (renal glomerulus, UBERON:0000074) are groups of capillaries that facilitate filtration of blood in the outer layer of kidney tissue known as the cortex^50^. The size of normal glomeruli in humans ranges from 100–350 μm in diameter, and they have a roughly spherical shape^6^. Glomeruli contain four cell types: parietal epithelial cells (CL:1000452), podocytes (CL:0000653), fenestrated endothelial cells (a.k.a. glomerular capillary endothelial cell CL:1001005), and mesangial cells (CL:1000742)^51^. Parietal epithelial cells form the Bowman’s capsule. Podocytes cover the outer layer of the filtration barrier. Fenestrated endothelial cells are in direct contact with blood and coated with a glycolipid and glycoprotein matrix called glycocalyx. Mesangial cells occupy the space between the capillary blood vessel loops and are stained by the colorimetric histological stain called Periodic acid-Schiff (PAS) stain^51^. PAS stains polysaccharides (complex sugars like glycogen) such as those found in and around the glomeruli making it a favored stain for delineating them in tissue sections^52^.

#### HuBMAP data

The kidney data used in the “HuBMAP - Hacking the Kidney” Kaggle competition comprises 30 whole slide images (WSIs) provided by the BIOmolecular Multimodal Imaging Center (BIOMIC) team at Vanderbilt University (VU) who are also members of HuBMAP’s Tissue Mapping Center at VU (TMC-VU). The tissue blocks were collected through the Cooperative Human Tissue Network^53^ and are either fresh frozen (FF) or formalin fixed, paraffin embedded (FFPE)^54^ for preservation. FF tissue is frozen in liquid nitrogen (−190 °C) within 30–60 min after surgical excision; this type of preservation has been the method of choice for transcriptomics and immunohistochemistry. Tissue samples are often embedded in Optimal Cutting Temperature (OCT) media for thin sectioning^55^ or carboxymethylcellulose (CMC) for imaging mass spectrometry^56^. FFPE tissue is the preferred method for clinical pathology samples for histology assessment since the formalin aldehyde cross-links proteins to maintain structural integrity of the sample^57^. After preservation, the tissue blocks were sectioned^58^ and imaged using PAS staining^59^. The slides were scanned with a brightfield scanner, and the resulting images were converted from vendor formats to Tagged Image File Format (TIFF). The images have an average size of 33,897 pixels (67,795 µm) by 31,469 pixels (62,938 µm) and a spatial resolution of 0.5 µm per pixel. On average, the 7102 glomeruli have an average area size of 80,498 pixels or 20,125 µm^2^.

Each of the 30 kidney datasets used in the Kaggle competition included a PAS stain WSI, anatomical region (AR) masks, and glomeruli segmentation masks. The masks were modified GeoJSON files that captured the polygonal outline of segmentations by their pixel coordinates (see samples in Fig. 2b). Initial segmentations were generated automatically by a segmentation pipeline^60^, then manually inspected and corrected by subject matter experts (SMEs)^61^ using QuPath^62^. In addition, information on sample size, location, rotation within the kidney, and pertinent clinical metadata (age, sex, ethnicity, BMI, laterality) was provided (see Supplementary Table 3).

For the Kaggle competition, this data was split into three datasets: public train (*n* = 15, for training models), public test (*n* = 5, for model validation), and private test (*n* = 10, for scoring and ranking models). The public datasets were openly available for the competitors to use when designing their models and creating submissions, and the private test set was only available to the Kaggle team and hosts for evaluation of the submissions. After the competition concluded, all data was made available publicly at the HuBMAP Data Portal^42^ as the “HuBMAP ‘Hacking the Kidney’ 2021 Kaggle Competition Dataset - Glomerulus Segmentation on Periodic acid-Schiff Whole Slide Images” collection^63^.

#### HPA data

Additional kidney data was collected from the HPA consisting of 99 kidney immunohistochemistry WSIs of 1 mm diameter tissue microarray cores and 4 µm thickness, stained with antibodies visualized with 3,3’-diaminobenzidine (DAB) and counterstained hematoxylin (H). The samples were taken from 8 donors (5 male and 3 female) between the ages 28 and 73. The 337 renal glomeruli in kidney WSIs were manually segmented by Subject Matter Experts (SMEs) using an internal web-based segmentation tool (developed by the HPA team and further modified by the HuBMAP team) and the standard operating procedure “SOP: Manual Segmentation of Tissue”^64^. All images are around 3000 × 3000 pixels, and the diameter of each tissue area within an image is around 2500 × 2500 pixels, which corresponds to 1 mm. Hence, the pixel size of the images in this dataset is around 0.4 µm. Metadata details are in Supplementary Table 5.

#### HuBMAP and HPA colonic crypts data

Colonic crypts (crypt of Lieberkuhn of large intestine, UBERON:0001984) are epithelial invaginations into the connective tissue (stroma) surrounding the colon or large intestine^65^. They contain stem/progenitor cells in their base and are thought to protect these cells from metabolites^66^. They are also the site of absorption and secretion activities within the colon^67^. Normal human colonic crypts have a diameter of 73.5 ± 3.4 µm and length of 433 ± 25 µm^68^. In addition to stem cells, there are many epithelial subtypes, and major subsets include: Paneth (CL:0009009), goblet (CL:1000321), enteroendocrine (CL:0000164), and enterocytes (CL:0002071)^65^. Total number of goblet cells increases from the proximal to distal ends of the colon^69^. Enterocytes are absorptive cells which decrease in number from the proximal to distal end of the colon and are responsible for absorption of nutrients^69^. Enteroendocrine cells make up a small proportion of the colonic epithelium (<1%) and secrete hormones that control gut physiology^69^.

#### HuBMAP data

The colon dataset was provided by the HuBMAP TMC-Stanford team. It consists of two TIFF WSIs and their GeoJSON annotations of colonic crypts. Each image is from a different donor and contains scans of four unique H&E-stained coverslips from different regions of the colon (ascending, transverse, descending, and descending sigmoid) for a total of 8 colon H&E images. One image was discarded as there were no crypts in it. Hematoxylin and eosin stain nucleic acids deep blue-purple and nonspecific proteins varying degrees of pink, respectively^70^. The two WSIs were annotated by Dr. Teri Longacre using QuPath^62^ and the “SOP: Manual Segmentation of Tissue”^64^. The resulting annotations were exported to GeoJSON format and included 389 individual crypt annotations, which on average had an area of 22,228 pixels, or 16.78 µm^2^, a considerably smaller average area than that of the glomeruli annotations (80,498 pixels, or 20,125 µm^2^); see Supplementary Table 4 for metadata.

#### HPA data

Similar to the HPA data for kidney, a dataset of 58 WSIs from the colon were sampled from the Human Protein Atlas. The samples were taken from 7 donors (3 male and 4 female) between the ages 47 and 84. Just like the HPA kidney data, the immunohistochemistry WSIs have a 1 mm diameter and 4 µm thickness and are stained with antibodies visualized with 3,3’-diaminobenzidine (DAB, looking brown) and counterstained hematoxylin (H). 3107 FTU segmentations were authored by colon experts. Metadata details are in Supplementary Table 5.

### Spatial location in human body

The HuBMAP Registration User Interface (RUI)^31,71^ was used to capture the three-dimensional size, position, and rotation of all tissue blocks in the HuBMAP data in close collaboration with subject matter domain experts. The resulting data was used to compute the vertical position of the mass points of all kidney tissue blocks as a proxy of the sequence of tissue sections. For the colon data, we report the location of tissue blocks according to the serial extraction sites (ascending colon, transverse colon, descending colon, sigmoid colon); see Fig. 2a.

### Computation of FTU density

The approximate number of glomeruli segmentations in a square millimeter of kidney cortex, henceforth referred to as “FTU density,” was calculated for HuBMAP data to compare it across cohorts of donors who varied in sex, age, race, and BMI. The 30 glomerulus annotation masks were read into a Jupyter Notebook (see utils directory in GitHub code repository) from *.json format and saved as Shapely polygons^72^. The average area per glomerulus per WSI was calculated in pixels, then converted to square micrometers. The anatomical region masks, which are rough estimates based on segmentations by SMEs, were read into the same Jupyter Notebook from *.json files as Shapely polygons, then the total cortex area per WSI was calculated by summing the area of all cortex segmentations, then converting from pixels to square micrometers. The approximate FTU density was calculated from these two values and converted to the number of glomeruli per square millimeter (see Supplementary Table 3).

### Postprocessing of predicted segmentation masks

In order to calculate precision and recall scores for the algorithms, overlapping or adjacent FTU segmentations must be separated into single FTUs.

#### HuBMAP data

The 70 predicted segmentation masks computed for the 14 WSIs by the five algorithms were manually examined, and FTUs that were overlapping or adjacent were separated via manual addition of a line.

In addition, we post-processed each segmentation mask using a watershed algorithm^73^ to separate overlapping or adjacent FTUs (see details in “Segmentation mask analysis” section). The results of this automated separation were close to the results from manual separation, except one colon image which showed drastic differences (see Slide 36 in Supplementary Tables 6 and 7 for details).

#### HPA data

The 780 predicted segmentation masks for the HPA data were not manually examined but processed only through the watershed pipeline to separate overlapping or adjacent FTUs.

### Kidney glomerulus segmentation prior work

For glomerulus segmentation, Sheehan et al. implemented a classifier trained on PAS stain murine renal images through Ilastik^14^. It performed well on their mouse validation set, but when applied to human data, it divided glomeruli and generated many false positives. Gallego et al. used transfer learning to fine tune the pre-trained AlexNet CNN with an overlapping sliding window method to segment and classify glomeruli in human WSIs of PAS-stained renal tissue. They discovered that the pre-trained model outperformed the model trained from scratch^7^. Govind et al. employed a Butterworth bandpass filter to segment glomeruli from multimodal images (autofluorescence and immunofluorescence marker stain)^74^. Kannan et al. also employed a CNN with an overlapping sliding window operator to segment glomeruli in trichrome-stained images, where they used training data of human origin and watershed segmentation^6^. Methods employing CNNs for the task of glomerulus segmentation seem to be increasingly popular in recent years with highly promising performance^8–10^. There has been much high-quality work done in the field of glomerular detection and segmentation through deep-learning methods, such as approaches that perform better for model species than humans^15^, focus on detecting disease states^16,17^, or focus on rapid FTU detection^18^. Recent work by Lutnick et al.^19^ uses deep-learning algorithms (CNN-based Deeplab-v2 architecture) for segmentation of glomeruli in histology images, using a dataset containing both human (healthy and diseased) and murine samples. It validates the success of CNN-based deep-learning algorithms on such tasks and uses the proprietary SVS/SCN image format in its processing pipeline. Details on algorithm type, performance metrics used, and scores achieved are in Supplementary Table 1.

### Colon crypt segmentation prior work

In 2010, Gunduz-Demir et al. approached the task of automatic segmentation of colon glands using an object-graph in conjunction with a decision tree classifier, which obtained a Dice coefficient of 88.91 ± 4.63, an improvement over the pixel-based counterparts at the time^75^. Five years later, Cohen et al. developed a memory-based active contour method that used a random-forest classifier that performed pixel-level classification with an F-measure of 96.2%^76^. That same year, the Gland Segmentation (GlaS) Challenge Contest was held in conjunction with the Medical Image Computing and Computer-Assisted Intervention (MICCAI 2015) convention^77^. Teams were challenged to present their solutions for automating segmentation of benign and malignant crypts within 165 images from 16 H&E-stained intestinal tissue sections, known as the Warwick-QU dataset. Chen et al. had the winning submission, dubbed “CUMedVision,” which was a novel deep-contour-aware, fully convolutional neural network (CNN)^78^. Kainz, Pfeiffer, and Urschler submitted the “vision4GlaS” method—a CNN for pixel-wise segmentation and classification paired with a contour-based approach to separate pixels into objects—to the GlaS Challenge Contest. Their method ranked 10th in the challenge’s entries^79^. They paired two distinct CNNs (Object-Net for predicting labels and Separator-Net for separating glands) together for pixel-wise classification of the same H&E-stained images^11^. For this second method, they also preprocessed the RBG images, only inputting the red channel into the model. Banwari et al. took a very computationally efficient approach to colonic crypt segmentation by also isolating the red channel from the GlaS Challenge dataset images and applying intensity-based thresholding^13^. Li et al. also used a portion of the GlaS Challenge dataset in 2016 to craft their model, a combination of a window-based classification CNN and hand-crafted features with support vector machines (HC-SVM)^80^. Sirinukunwattana, Snead, and Rajpoot used the GlaS challenge dataset in 2015 to develop a random-polygons model^81^. In 2018, Tang, Li, and Xu’s Segnet model for crypt segmentation outperformed the contest winner in some portions of the challenge^82^. One of the most recent uses of the GlaS Challenge dataset was by Graham et al. in 2019 for the development of their Minimal Information Loss Dilated Network (MILD-Net) segmentation method which performs simultaneous crypt and lumen segmentation. Their proposed network “counters the loss of information caused by max-pooling by re-introducing the original image at multiple points within the network” and received higher evaluation metric scores than the winner of the GlaS Challenge or Segnet^83^. Another use of the GlaS Challenge dataset was by Rathore et al. as they tested the efficacy across institutions of their support vector machine (SVM) method for segmenting colonic crypts^84^. Details on algorithm type, performance metrics used, and scores achieved are in Supplementary Table 2.

### Competition design

The “HuBMAP - Hacking the Kidney” Kaggle competition challenged teams to develop machine-learning algorithms to segment renal glomeruli FTUs in kidney histopathological images across different tissue preparation pipelines (FF and FFPE). The goal was to develop a highly accurate and robust FTU segmentation algorithm.

Two separate types of prizes were offered: Accuracy Prizes and Judges Prizes. The Accuracy Prize awarded $32,000 to the three teams with the highest scores on the Kaggle leaderboard at the conclusion of the competition (1st: $18,000, 2nd: $10,000, 3rd: $4000). The Judges Prize awarded $28,000 to the teams that advanced science and/or technology (Scientific Prize: $15,000), were the most innovative (Innovation Prize: $10,000), or were the most diverse (Diversity Prize: $3000) as identified by the panel of judges through a presentation of the teams’ findings and subsequent scoring based on a predetermined rubric^85^. The complete evaluation rubric, as presented below, was used by the judges to evaluate the winners in each category. Each criterion consists of ten points, for a total of 50 points for scientific prize, 30 points for innovation prize, and 30 points for diversity prize. The criteria for the scientific prize was:

1. Are the statistical and modeling methods used to identify glomeruli in the PAS-stained microscopy data appropriate for the task?
2. Are confidence scores and other metrics provided that help interpret the results achieved by the modeling methods?
3. Did the team validate their methods and algorithm implementations and provide information on algorithm performance and limitations?
4. Did the team provide any evidence that their method generalizes beyond this immediate task—for example, to other FTUs such as alveoli in lungs or crypts in colon?
5. Did the team document their method and code appropriately?

The criteria for the innovation prize was:

1. Did the team develop a creative or novel method to segment glomeruli?
2. Is the presented characterization of glomeruli useful for understanding individual differences—e.g., the impact of donor sex, age, or weight on the size, shape, or spatial distribution of glomeruli?
3. Did the team provide insights that would be useful for generating reference glomeruli for inclusion into the HRA?

The criteria for the diversity prize was:

1. Does the team embrace diversity and equity, welcoming team members of different ages, genders, ethnicities, and with multiple backgrounds and perspectives?
2. Did the authors effectively communicate the details of their method for segmenting glomeruli, and the quality and limitations of their results? For example, did they use data visualizations to present algorithm setup, run, results, and/or to provide insight into the comparative performance of different methods? Were these visualizations effective at communicating insights about their approach and results?
3. Are the important results easily understood by the average person?

Teams were allowed to enter in multiple categories and had the option of either receiving cash prizes or choosing to have their winnings donated to a charity foundation. In addition, the use of supplemental publicly available training data was allowed, under the condition that the data would be shared with other participants on the Kaggle discussion board to maintain fairness of data availability. The organizers and judges were not permitted to participate.

The competition launched on November 16, 2020, and ran through the final submission date of May 10, 2021. The data was updated and timeline extended on March 9, 2021. The Awards ceremony was held on May 21, 2021. Submissions were made in the form of Kaggle notebooks with a run-length encoding of the predictions saved in a submission.csv file. The notebooks had to run in less than or equal to 9 hours without internet access. See Competition Rules^86^ and Judging Rubric^85^ for more details.

Algorithm performance was evaluated using the mean Dice coefficient (see “Metrics”). The leaderboard scores were the mean of the Dice coefficients for all ten WSIs in the private test set. Any test WSI with predictions missing completely were factored into the mean score as a zero. This metric is a popular metric to test segmentation performance and has been successfully used for previous segmentation task challenges. For example, 922 teams competed in the “Ultrasound Nerve Segmentation” Kaggle competition^87^. The top scoring teams achieved a mean Dice coefficient of 0.7323 and 0.7313 for the private and public leaderboards, respectively. Another competition, entitled “SIIM-ACR Pneumothorax,” engaged 1475 teams to classify and segment pneumothorax from chest radiographic images, with leaderboard scores topping at 0.8679 and 0.9304 mean Dice coefficients for private and public datasets, respectively^88^. A third competition,”Severstal: Steel Defect Detection,” focused on localizing and classifying surface defects on a sheet of steel^89^; it had 2427 teams competing and achieved mean Dice coefficients of 0.9088 (private leaderboard) and 0.9247 (public leaderboard).

In the “HuBMAP - Hacking the Kidney” Kaggle competition, a total of 1200 teams competed and the top-5 scoring teams had a mean Dice coefficient of 0.9515 and 0.9512 for the private and public leaderboards, respectively.

### Training strategies and transfer learning

While the Kaggle competition involved developing models for segmenting glomeruli in kidney tissue samples, it is crucial to test the generalization capability of such segmentation models across organs. To accomplish this goal, we implemented several strategies to train models and run inference for the models using test data. Figure 1 provides an overview of data sources, the splitting of data into test, train, and validation datasets for the 5 strategies, and the results of the best-performing algorithm in terms of the mean Dice value. Strategy 1 trains the models on the HuBMAP kidney data and tests them on the HuBMAP kidney data. Strategy 2 trains the five models on HuBMAP colon data (with no pretraining on HuBMAP kidney data) and tests them on HuBMAP colon data. Strategy 3 uses models pretrained on the HuBMAP kidney data (see Strategy 1), trains them further on the HuBMAP colon data, and tests them on the HuBMAP colon data; this is a type of transfer learning (i.e., using models trained on kidney data as initial weights for training on colon data). Strategy 4 trains models on the HuBMAP kidney data (see Strategy 1) and tests them on HPA kidney data. Strategy 5 trains models on the HuBMAP kidney and colon data (see Strategy 3 training) and tests them on the HPA colon data.

Model training in Strategy 3 is called “transfer learning” in machine learning. It is a widely used technique to improve performance on a dataset by pretraining it on a different but similar dataset. Transfer learning allows a model to learn features from an existing dataset and helps improve the generalizability of the overall model. This is especially useful in cases where training data is insufficient. In this paper, ‘transfer learning’ refers to training a ML model using kidney data so it can segment glomeruli and then using this pretrained model as initial weights for training on a different but related task, e.g., to segment crypts in colon data. That is, knowledge gained while learning to recognize a glomeruli is applied when trying to recognize crypts. Note that transfer learning may involve training the entire pretrained model or freezing some layers of the pretrained model and further training the remaining unfrozen layers. It should be noted that all models used in the paper are already pretrained on the Imagenet dataset^90^ that comprises 14,197,122 real-world images.

### Algorithms

Teams Tom, Gleb, and Whats goin on won first, second, and third place for the accuracy prize, respectively. DeepLive.exe and Deepflash2 won the first (scientific prize) and second (innovation) judges prizes, respectively. The setup, optimization, and prediction run of all five algorithms are discussed here.

#### Tom

The model reads the WSIs as tiled 1024 × 1024 pixel images, resizing them to 320 × 320 tiles which are sampled using a balanced sampling strategy. It is trained for 20 epochs (iterations), with a learning rate of 10^−4^ to 10^−6^ and a batch size of 8 (i.e., training is done using batches of 8 images per batch).

In addition, for the model trained on the HuBMAP colon data (with and without pretraining on HuBMAP kidney data), the training is done for 50–100 epochs and the validation set is increased from 1 slide to 2 slides.

#### Gleb

The models read tiles of size 1024 × 1024 sampled from the HuBMAP kidney/colon data. During model training, general data augmentation techniques such as adding gaussian blur and sharpening, adding gaussian noise, and applying random brightness or gamma value are used. The models are trained for 50–80 epochs each, with a learning rate of 10^−4^ to 10^−6^ and a batch size of 8.

In addition, the model trained on the HuBMAP colon data (with and without pretraining on HuBMAP kidney data) is trained for 50–100 epochs, and the sampling downscale factor is changed from 3 to 2.

#### Whats goin on

The model downsamples the input images by a factor of 2 and tiles of size 1024 × 1024 are sampled followed by filtering based on a saturation threshold of 40. General data augmentation techniques are used such as flipping, rotation, scale shifting, deformation, artificial blurring, Hue Saturation Value (HSV) shifting, Contrast Limited Adaptive Histogram Equalization (CLAHE), brightness and contrast shifting, and Piecewise Affine. The models are trained for 50 epochs each, using a one-cycle learning-rate scheduler with specific hyperparameters set as pct_start = 0.2, div_factor = 1e2, max_lr = 1e−4, and batch size of 16. The models use an expansion tile size of 32.

In addition, for the model trained on the HuBMAP colon data (with and without pretraining on the HuBMAP kidney data), the batch size is increased to 64 and the expansion tile size is increased to 64.

#### DeepLive.exe

Model training uses an adaptive learning rate, which is linearly increased up to 0.001 during the first 500 iterations and then linearly decreased to 0, and a batch size of 32. During training, general data augmentation techniques are used such as brightness and contrast changes, RGB shifting, HSV shifting, color jittering, artificial blurring, CutMix^91^, and MixUp^92^. The model is trained using 5-fold cross validation for at least 10,000 iterations.

In addition, for the model trained on the HuBMAP colon data (without pretraining on kidney data), on_spot_sampling of 1 and an overlap factor of 2 is used. For the model trained on HuBMAP colon data (pretrained on kidney data), on_spot_sampling is set to 1 and an overlap factor of 1 is used. In both cases, no external datasets are used for training.

#### Deepflash2

On runtime, the model samples tiles of size 512 × 512 and uses a resolution downscale factor of 2, 3, and 4 in subsequent runs. During training, general data augmentation techniques are applied such as flipping, blurring, deformation, etc. Model training uses a maximum learning rate of 1e−3, and a batch size of 16. The model training is done using a learning-rate scheduler whereby the learning rate is scheduled with a cosine annealing^93^ from max_learning_rate/div to max_learning_rate (where div = 25). The models are trained and tested using 5-fold cross validation in which each fold is trained on 12 WSIs and validated on 3 WSIs.

For the model trained on the colon data (both with and without pretraining on kidney data), the background probability is set to 0.1 and the colon probability is set to 0.9 for sampling, since the colon data lacks the masks for anatomical structures. A weight decay of 10^−5^ was added (for the model trained without transfer learning). For Strategy 3, saved weights are loaded from the model pre-trained on kidney data at 3x downsampling and the first 13 parameter groups are frozen during training.

For all five models, inference on the HPA kidney data is done using the final model trained on HuBMAP kidney data, and inference on the HPA colon data is done using the final model trained on HuBMAP colon data (pretrained on HuBMAP kidney data). The hyperparameter values used for training the models on the HuBMAP kidney data are the same values used in the competition by the original creators of the models.

### Performance metrics terminology

#### Ground Truth

**Fig. 5:**
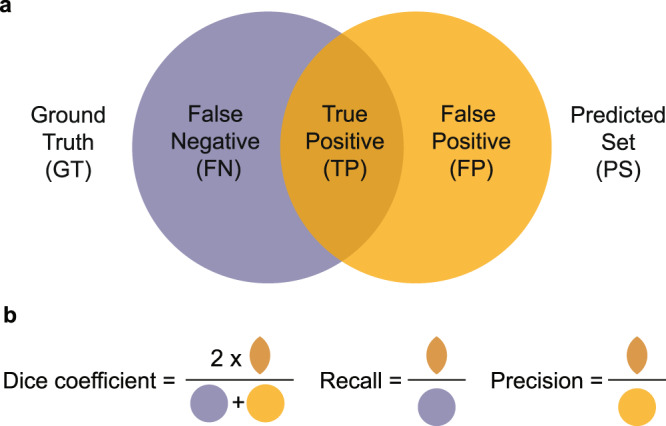
Performance metrics terminology. **a** Datasets: ground truth, predicted set, and false negatives, true positives and false positives. **b** Metrics: Dice coefficient, recall, and precision.

The set of all FTU segmentations in the human annotated dataset is called ground truth (GT, blue in Fig. 5a).

#### Predicted set

The set of all FTU segmentations predicted by an algorithm is called the predicted set (PS, yellow in Fig. 5a).

#### False negatives, true positives, and false positives

Typically, the GT and PS sets overlap creating three sets that are called false negatives (FN, FTUs not predicted by the algorithm), true positives (TP, FTUs in ground truth that are correctly predicted by the algorithm), and false positives (FP, FTUs predicted by the algorithm but not present in the ground truth); see Fig. 5a.

The sets can be represented via vector-based polylines or pixel masks, and different algorithms are used to compare these. Note that the metrics in Fig. 5b can be applied to pixels that represent an object of interest (e.g., an FTU) or to FTU counts.

### Performance metrics

Dice coefficient, or Sørensen–Dice index^94^, is widely used to compare the pixel-wise agreement between a predicted segmentation and its corresponding ground truth. The formula is given by $\frac{2* {|GT}\cap {PS|}}{{|GT|}+{|PS|}}$ (see Fig. 5b). The Dice coefficient is defined to be 1 when both sets are empty.

Mean Dice coefficient is the sum of all Dice coefficients (e.g., one for each image in the test set) divided by the count of all numbers in the collection (e.g., the number of images in the test set).

Recall, also referred to as sensitivity, measures the proportion of instances that were correctly predicted compared to the sum of false negatives and true positives. It is defined as $\,\frac{{TP}}{{TP}+{FN}}$ (see Fig. 5b).

Precision denotes the proportion of predictions that were correct and is defined as $\,\frac{{TP}}{{TP}+{FP}}$ (see Fig. 5b).

Other performance metrics used by related work; see Supplementary Tables 1 and 2:

#### F-measure/F-score/F_1_-score

The F-measure, also called the F-score or F_1_-score is the harmonic mean of Precision and Recall, defined as $\frac{2* {Precision}* {Recall}}{{Precision}+{Recall}}$.

#### Accuracy

Accuracy is the proportion of correct predictions as defined by $\,\frac{{TP}+{TN}}{{TP}+{TN}+{FP}+{FN}}$.

#### Matthews correlation coefficient

The Matthews correlation coefficient is used for binary classifiers to provide a balanced measure of quality^6^. It is defined as $\,\frac{{TP}* {TN}-{FP}* {FN}}{\sqrt{({TP}+{FP})({TP}+{FN})({TN}+{FP})({TN}+{FN})}}$.

#### Hausdorff distance

The Hausdorff distance is a measure used to calculate how similar two objects or images are to one another by calculating the distance between two sets of edge points^95^.

#### Jaccard index

The Jaccard index, also known as Intersection over Union (IoU), is defined by $J(A,B)=\frac{{|A}\cap {B|}}{{|A}\cup {B|}}=\frac{{|A}\cap {B|}}{{|A|}+{|B|}-{|A}\cap {B|}}$, where *A* and *B* are the two objects being compared (e.g., GT and PS in Fig. 5a). It represents the proportion of area of overlap out of the area of union for the two objects.

### Segmentation mask analysis

Ground truth segmentation masks were provided as vector files (one polyline per FTU; many FTUs per WSI). However, algorithm predictions are generated as run-length encodings—one mask for all FTUs in each WSI. Some FTUs are adjacent, effectively merging multiple FTUs into one; this makes it impossible to count the predicted FTUs and compute the Dice coefficient per FTU but also recall and precision per FTU.

To separate overlapping or adjacent FTUs, we manually added 647 lines to the 70 predicted HuBMAP WSI segmentation masks (232 lines for 50 kidney slides and 415 lines for 20 colon slides). We then converted pixel masks for each FTU into one polyline per FTU. Next, we calculated the Dice coefficient for each segmented FTU (glomerulus or crypt) separately; assuming that a Dice coefficient greater or equal than 0.5 indicates that the FTU was correctly predicted, the set of true positives. All FTUs with a Dice coefficient less than 0.5 are false positives (FP), while all ground truth masks with no matching algorithm predictions are false negatives (FN). All results of Dice coefficient, recall, and precision computations are provided in Supplementary Table 6 which lists Dice coefficients, false negatives (FN), true positives (TP), and false positives (FP) of winning algorithms for individual WSIs in all three predicted datasets (10 kidney WSIs using Strategy 1; 2 colon WSIs using Strategy 2; 2 colon WSIs using Strategy 3, see Fig. 1 for an explanation of all strategies).

In addition, we processed each segmentation mask using a watershed algorithm (a classical algorithm used to separate different objects in an image) considerably speeding up the process. Specifically, we used scikit-image’s specific implementation of watershed^96^. The algorithm uses a tunable minimum distance parameter. A minimum distance of 30 and 60 is used for processing the kidney data and colon data, respectively. The results of this automated separation process were close to the results from manual separation, except one colon image which showed drastic differences.

The 780 predicted segmentation masks for the HPA data were not manually examined and instead were processed only through the watershed pipeline to separate the FTU instances. A minimum distance of 80 was used for processing the kidney data and colon data.

### Statistics and reproducibility

The analysis for the computation of FTU density across donors was conducted using Jupyter Notebooks, coded in the Python programming language, and data was formatted as *.json files (see “Computation of FTU density” under Methods). The analysis used a total of 16 donors (8 male and 8 female). The data for Fig. 3 and Fig. 4 was compiled as .txt files, each file containing the precision and recall values for each glomeruli and crypt. The source data files are provided as part of the data (see “Data availability”). All data used in this study is secondary data which was already collected as part of the HuBMAP and HPA projects hence, no study protocol was used as it was not applicable to this data or study. All available donor metadata for all WSIs used is provided in Supplementary Tables 3, 4, and 5.

### Reporting summary

Further information on research design is available in the Nature Portfolio Reporting Summary linked to this article.

## Supplementary information

Supplementary Information

Reporting Summary

## Data Availability

Kidney data used in the Kaggle competition has been published via the HuBMAP portal as a collection at 10.35079/hbm925.sgxl.596. All data (HuBMAP and HPA, with ground truth masks and predictions, source data for Figs. 3 and 4) are available as a Zenodo dataset at 10.5281/zenodo.7729609. All trained models are available as a Zenodo dataset at 10.5281/zenodo.7730027.

## Code availability

Code to re-run the algorithm comparison, implementation of watershed algorithm, compute performance metrics, and reproduce figures is available at https://github.com/cns-iu/ccf-research-kaggle-2021. The version of code at the time of publication is also available on Zenodo at 10.5281/zenodo.7730067. An interactive companion website is available at https://cns-iu.github.io/ccf-research-kaggle-2021/.
