# Supplementary material for: Segmentation of human functional tissue units in support of a Human Reference Atlas
Source: Commun Biol. 2023 Jul 19;6:717. doi: 10.1038/s42003-023-04848-5 (PMC10356924; doi:10.1038/s42003-023-04848-5)
Supplement: Supplementary file 1 — Supplementary Information [file 42003_2023_4848_MOESM1_ESM.pdf]

Supplementary Information for Segmentation of Human Functional Tissue Units in Support of a Human Reference Atlas

Authors

Yashvardhan Jain[1*], Leah L. Godwin[1], Yingnan Ju[1], Naveksha Sood[1], Ellen M. Quardokus[1], Andreas Bueckle[1], Teri Longacre[2], Aaron Horning[3], Yiing Lin[4], Edward D. Esplin[5], John W. Hickey[6], Michael P. Snyder[5], Nathan Heath Patterson[7], Jeffrey M. Spraggins[7,8], Katy Börner[1]*

[1]Department of Intelligent Systems Engineering, Luddy School of Informatics, Computing, and Engineering, Indiana University, Bloomington, IN 47408, USA

[2]Department of Pathology, Stanford University School of Medicine, Stanford, CA 94305, USA

[3]Thermo Fisher Scientific, South San Francisco, CA 94080, USA

[4]Department of Surgery, Washington University School of Medicine, St. Louis, MO 63110, USA

[5]Department of Genetics, Stanford University School of Medicine, Stanford, CA 94305, USA

[6]Department of Microbiology & Immunology, Stanford University School of Medicine, Stanford, CA 94305, USA

[7]Mass Spectrometry Research Center, Vanderbilt University, Nashville, TN 37232, USA

[8]Department of Cell and Developmental Biology, Vanderbilt University, Nashville, TN 37232, USA

*Corresponding authors

Yashvardhan Jain yashjain@iu.edu

Katy Börner katy@indiana.edu

Supplementary Tables

Supplementary Table 1. Prior work on renal glomerulus segmentation. This table lists prior work on renal glomerulus segmentation. For each published paper given in the Reference column, we list model name (if applicable), algorithm type, tissue donor species, performance metrics used, and scores achieved.

Supplementary Table 1. Prior work on renal glomerulus segmentation

Reference	Model Name	Algorithm Type	Species	Performance Metric(s)	Score(s)
Sheehan, S. M. & Korstanje, R. Automatic glomerular identification and quantification of histological phenotypes using image analysis and machine learning. Am. J. Physiol. - Ren. Physiol. 315, F1644–F1651 (2018).	-	Ilastik object classifier	Mouse	Precision, Recall, F-measure	0.984, 0.952, 0.960
			Rat	Precision, Recall	0.523, 0.986
			Human	Recall	0.89
Bukowy, J. D. et al. Region-Based Convolutional Neural Nets for Localization of Glomeruli in Trichrome-Stained Whole Kidney Sections. J. Am. Soc. Nephrol. 29, 2081–2088 (2018).	-	Faster RCNN	Rat	Precision, Recall	0.9694, 0.9679
			Human	Precision, Recall	0.802, 0.8167
Gallego, J. et al. Glomerulus Classification and Detection Based on Convolutional Neural Networks. J. Imaging 4, 20 (2018).	-	AlexNet CNN	Human	F-measure	0.937
Govind, D., Ginley, B., Lutnick, B., Tomaszewski, J. E. & Sarder, P. Glomerular detection and segmentation from multimodal microscopy images using a Butterworth band-pass filter. in Medical Imaging 2018: Digital Pathology vol. 10581 1058114 (International Society for Optics and Photonics, 2018).	-	Butterworth band-pass filter	Mouse	Accuracy, Error, F-measure, Recall, Specificity, Precision	0.8731, 0.13, 0.83, 0.95, 0.84, 0.74
Kannan, S. et al. Segmentation of Glomeruli Within Trichrome Images Using Deep Learning. Kidney Int. Rep. 4, 955–962 (2019).	-	Google's Inception v3 CNN	Human	Specificity, Recall, F-measure, Matthews correlation coefficient	0.999, 0.558, 0.623, 0.628
Pedraza, A. et al. Glomerulus Classification with Convolutional Neural Networks. in Medical Image Understanding and Analysis (eds. Valdés Hernández, M. & González-Castro, V.) 839–849 (Springer International Publishing, 2017). doi: 10.1007/978-3-319-60964-5_73.	-	Pre-trained AlexNet CNN	Human	F-measure	0.999
Hermsen, M. et al. Deep Learning–Based Histopathologic Assessment of Kidney Tissue. J. Am. Soc. Nephrol. 30, 1968–1979 (2019).	-	U-net CNN	Human	Dice coefficient	0.95
Marsh, J. N. et al. Deep Learning Global Glomerulosclerosis in Transplant Kidney Frozen Sections. IEEE Trans. Med. Imaging 37, 2718–2728 (2018).	-	Pre-trained VGG16 CNN	Human	Precision, Recall, F-measure	0.932, 0.962, 0.947
Ginley, B. et al. Computational Segmentation and Classification of Diabetic Glomerulosclerosis. J. Am. Soc. Nephrol. 30, 1953–1967 (2019).	-	Google's Deeplab CNN	Human+ Mice	Balanced Accuracy, Sensitivity, Specificity	0.93, 0.88, 0.9995

Supplementary Table 2. Prior work on colon crypts segmentation. This table lists prior work on colon crypts segmentation. For each published paper given in the Reference column, we list model name (if applicable), algorithm type, tissue donor species, performance metrics used, and scores achieved.

Supplementary Table 2. Prior work on colon crypts segmentation

Reference	Model Name	Algorithm Type	Subject species	Performance Metric(s)	Score(s)
Gunduz-Demir, C., Kandemir, M., Tosun, A. B. & Sokmensuer, C. Automatic segmentation of colon glands using object-graphs. Med. Image Anal. 14, 1–12 (2010).	-	Object-graph + decision tree classifier	Human	Recall,<br>Specificity,<br>Accuracy,<br>Dice coefficient	0.8580 ± 0.671,<br>0.8914 ± 0.1040,<br>0.8759 ± 0.501,<br>0.8891 ± 0.463
Cohen, A., Rivlin, E., Shimshoni, I. & Sabo, E. Memory based active contour algorithm using pixel-level classified images for colon crypt segmentation. Comput. Med. Imaging Graph. 43, 150–164 (2015).	-	Memory Based Active Contour Algorithm: Pixel classifier + active contour	Human	Recall,<br>Accuracy,<br>F-measure	0.87,<br>0.96,<br>0.962
Chen, H., Qi, X., Yu, L. & Heng, P.-A. DCAN: Deep Contour-Aware Networks for Accurate Gland Segmentation. ArXiv160402677 Cs (2016).	"CUMedVision" /DCAN	Deep Contour-Aware Network	Human	F-measure,<br>Dice coefficient,<br>Hausdorff distance	0.9116,<br>0.8974,<br>45.4182
Kainz, P., Pfeiffer, M. & Urschler, M. Segmentation and classification of colon glands with deep convolutional neural networks and total variation regularization. PeerJ 5, e3874 (2017). / Kainz, P., Pfeiffer, M. & Urschler, M. Semantic Segmentation of Colon Glands with Deep Convolutional Neural Networks and Total Variation Segmentation. ArXiv151106919 Cs (2017).	"vision4GlaS"	Two deep CNNs: pixel classifier + contour based	Human	Precision,<br>Recall,<br>F-measure,<br>Dice coefficient,<br>Hausdorff distance	0.67,<br>0.77,<br>0.68,<br>0.75,<br>103.49
Banwari, A., Sengar, N., Dutta, M. K. & Travieso, C. M. Automated segmentation of colon gland using histology images. in 2016 Ninth International Conference on Contemporary Computing (IC3) 1–5 (2016). doi:10.1109/IC3.2016.7880223.	-	Intensity based thresholding	Human	Accuracy	0.9376
Li, W. et al. Gland segmentation in colon histology images using hand-crafted features and convolutional neural networks. in 2016 IEEE 13th International Symposium on Biomedical Imaging (ISBI) 1405–1408 (2016). doi:10.1109/ISBI.2016.7493530.	-	Hand-crafted Support Vector Machine + Alexnet CNN	Human	Jaccard index,<br>Dice coefficient	0.77 ± 0.11,<br>0.87 ± 0.08
Sirinukunwattana, K., Snead, D. R. J. & Rajpoot, N. M. A Stochastic Polygons Model for Glandular Structures in Colon Histology Images. IEEE Trans. Med. Imaging 34, 2366–2378 (2015).	Stochastic Polygons Model	Random Polygons Model (RPM)	Human	Jaccard index,<br>Dice coefficient,<br>Execution time (seconds)	0.74 ± 0.11,<br>0.82 ± 0.09,<br>206.4 ± 332.5
Tang, J., Li, J. & Xu, X. Segnet-based gland segmentation from colon cancer histology images. in 2018 33rd Youth Academic Annual Conference of Chinese Association of Automation (YAC) 1078–1082 (2018). doi: 10.1109/YAC.2018.8406531.	"Segnet"	CNN with pixel-wise classifier	Human	Dice coefficient,<br>Hausdorff distance	0.8636,<br>102.5729
Graham, S. et al. MILD-Net: Minimal Information Loss Dilated Network for Gland Instance Segmentation in Colon Histology Images. Med. Image Anal. 52, 199–211 (2019).	"MILD-Net"	CNN with MIL unit	Human	F-measure,<br>Dice coefficient,<br>Hausdorff distance	0.844,<br>0.836,<br>105.89
Rathore, S. et al. Segmentation and Grade Prediction of Colon Cancer Digital Pathology Images Across Multiple Institutions. Cancers 11, 1700 (2019).	-	SVM	Human	Accuracy,<br>Jaccard index,<br>Dice coefficient,<br>Recall,<br>Specificity,<br>F-measure	0.8840,<br>0.89,<br>0.87,<br>0.92,<br>0.88,<br>0.89

Supplementary Table 3. HuBMAP kidney metadata. This table provides metadata for all 30 kidney WSIs, one per row. For each WSI, we assigned a running number ID that is also used in **Fig. 2** and **5**. We provide corresponding HuBMAP sample and donor IDs, as well as the Kaggle IDs. We list tissue preservation method (fresh frozen, FF; formalin fixed, paraffin embedded, FFPE), image width and height in pixels, race (White, W; Black or African American, B), sex (male, M; female, F), weight, height, BMI, age, laterality (right kidney, R; left kidney, L), tissue block vertical location (y-position) according to Registration User Interface (RUI) registration. We also computed the glomerulus annotation area in square micrometers and the approximate number of glomeruli per square millimeter of kidney cortex.

Note that there is one patient with 4 tissue blocks in this dataset, 3 patients with 3 blocks, 5 patients who have 2, and the rest only have one. Of the 30 tissue blocks, 25 are from White patients and 5 are from Black or African American patients. The dataset is evenly divided between Male/Female sources. All females sampled were White, but male samples were split between White (10) and Black or African American (5). None of the samples were associated with a Hispanic or Latino ethnicity. All samples came from adults (minimum age 31 years old). The average weight (85.9kg) lies between the average weights for females (77.47kg) and males (90.63kg) in the United States[97]. The average height (170.35cm) also lies between the average heights for females (161.29cm) and males (175.26cm) in the US[97]. The average BMI of the dataset (29.61kg/m^2) is between that given as the average for females (29.8kg/m^2) and males (29.4kg/m^2) in the US[97]. Only 6 of the samples fell into the "Healthy weight" category

(18.5–24.9), and they originated from two patients. The other 24 samples were either in the "Overweight" (25.0–29.9, 8 samples) or "Obese" (30 and above, 16 samples) categories. There are no noticeable abnormalities when comparing weight and height between the sexes. Average BMI was still above "healthy" (24.9) for each subset when sex was taken into account.

Supplementary Table 3. HuBMAP kidney metadata

Slide	HuBMAP ID	Donor ID	Kaggle ID	Tissue preservation method	Image width (pixels)	Image height (pixels)	Race	Sex	Weight (kg)	Height (cm)	BMI	Age	Laterality	Tissue block y-position in 3D reference organ	Number of glomeruli	Average glomerulus area (pixels)	Average glomerulus area (μm^2)	Approximate number of glomeruli per square mm cortex
1	HBM874.RZDW.757	HBM679.GXQW.326	095bf7a1f	FF	39000	38160	W	F	71.7	160	28	44	R	11.08	350	99,420	24,855	2.39
2	HBM463.JRTB.582	HBM938.LVRS.434	aa05346ff	FF	47340	30720	W	F	59	160	23	58	R	11.69	325	116,612	29,153	2.06
3	HBM636.ZPTS.368	HBM938.LVRS.434	b9a3865fc	FFPE	40429	31295	W	F	59	160	23	58	R	13.70	469	56,324	14,081	4.22
**4**	**HBM324.ZGZM.874**	**HBM485.HTBW.247**	**e464d2f6c**	**FFPE**	**40816**	**50560**	**W**	**F**	**91.6**	**165.1**	**33.6**	**57**	**R**	**13.93**	**315**	**91,096**	**22,774**	**1.96**
**5**	**HBM832.FQKR.463**	**HBM485.HTBW.247**	**bacb03928**	**FF**	**22163**	**23968**	**W**	**F**	**91.6**	**165.1**	**33.6**	**57**	**R**	**14.11**	**118**	**56,384**	**14,096**	**2.74**
**6**	**HBM649.XFQG.775**	**HBM485.HTBW.247**	**ff339c0b2**	**FFPE**	**38912**	**48544**	**W**	**F**	**91.6**	**165.1**	**33.6**	**57**	**R**	**14.28**	**341**	**90,088**	**22,522**	**2.34**
7	HBM958.GHFM.676	HBM938.LVRS.434	afa5e8098	FF	43780	36800	W	F	59	160	23	58	R	16.35	235	113,048	28,262	1.63
**8**	**HBM296.RLWW.755**	**HBM485.HTBW.247**	**a14e495cf**	**FFPE**	**32768**	**62688**	**W**	**F**	**91.6**	**165.1**	**33.6**	**57**	**R**	**20.31**	**355**	**83,784**	**20,946**	**2.29**
9	HBM623.RPMC.638	HBM455.HLHM.985	3589adb90	FFPE	22165	29433	W	F	71.3	167.6	25.4	66	L	5.91	239	55,472	13,868	4.93
10	HBM276.PGFS.693	HBM769.HVDR.369	2f6ecfcdf	FFPE	25794	31278	W	F	93	157.4	37.5	76	L	9.58	160	49,820	12,455	3.27
11	HBM849.XMPC.398	HBM455.HLHM.985	26dc41664	FF	42360	38160	W	F	71.3	167.6	25.4	66	L	10.81	245	105,764	26,441	2.57
12	HBM362.PTQJ.743	HBM758.JRSC.348	d488c759a	FF	29020	46660	W	F	81.5	158.8	32.2	66	L	11.15	175	83,864	20,966	2.08
**13**	**HBM673.JJRZ.435**	**HBM633.KPHW.963**	**5274ef79a**	**FF**	**18491**	**22134**	**W**	**F**	**74.6**	**162.6**	**28.2**	**77**	**L**	**12.42**	**51**	**47,564**	**11,891**	**1.92**
14	HBM979.HDZH.896	HBM769.HVDR.369	57512b7f1	FF	43160	33240	W	F	93	157.4	37.5	76	L	18.72	141	97,408	24,352	1.59
15	HBM875.QHDJ.259	HBM547.NCQL.874	aaa6a05cc	FFPE	13013	18484	W	F	87.5	162.3	33.2	73	L	99.49	99	43,340	10,835	4.52
**16**	**HBM344.LLLV.539**	**HBM429.BVWN.357**	**5d8b53a68**	**FF**	**36732**	**22153**	**W**	**M**	**116.9**	**182.9**	**34.9**	**62**	**R**	**9.75**	**320**	**58,700**	**14,675**	**3.2**
**17**	**HBM627.RSGW.898**	**HBM226.XVDP.877**	**00a67c839**	**FFPE**	**28672**	**30400**	**W**	**M**	**96.6**	**175.3**	**31.4**	**66**	**R**	**14.02**	**97**	**91,440**	**22,860**	**1.16**
**18**	**HBM227.THVC.544**	**HBM226.XVDP.877**	**0749c6ccc**	**FFPE**	**26624**	**30368**	**W**	**M**	**96.6**	**175.3**	**31.4**	**66**	**R**	**14.18**	**109**	**91,716**	**22,929**	**1.55**
19	HBM783.GJWP.694	HBM368.WSHR.356	0486052bb	FFPE	34937	25784	W	M	106.1	180.3	32.6	31	R	14.33	130	73,324	18,331	2.51
20	HBM783.GDKK.879	HBM522.WZBV.379	1e2425f28	FF	32220	26780	W	M	131.5	193	35.3	48	R	18.05	178	104,856	26,214	1.89
21	HBM833.DBGG.252	HBM322.KQBK.747	2ec3f1bb9	FFPE	47723	23990	W	M	91.2	167.6	32.5	56	L	50.70	399	56,536	14,134	2.85
22	HBM389.MBWW.346	HBM745.MDSR.597	e79de561c	FF	27020	16180	B	M	73	166	26.5	53	L	51.24	180	88,100	22,025	2.6
**23**	**HBM649.DLZF.463**	**HBM322.KQBK.747**	**1eb18739d**	**FF**	**33103**	**20329**	**W**	**M**	**91.2**	**167.6**	**32.5**	**56**	**L**	**54.63**	**157**	**65,540**	**16,385**	**2.49**
24	HBM662.PMPZ.644	HBM322.KQBK.747	c68fe75ea	FF	19780	26840	W	M	91.2	167.6	32.5	56	L	56.86	118	110,656	27,664	0.92
25	HBM879.CDHB.995	HBM745.MDSR.597	b2dc8411c	FFPE	31262	14844	B	M	73	166	26.5	53	L	57.83	138	44,232	11,058	4.05
**26**	**HBM984.PMZN.942**	**HBM525.JNPV.685**	**9e81e2693**	**FF**	**33100**	**27642**	**B**	**M**	**79.9**	**190.5**	**22**	**58**	**L**	**61.97**	**175**	**102,676**	**25,669**	**2.35**
27	HBM264.XSVF.528	HBM687.KPKM.763	4ef6695ce	FF	50680	39960	W	M	91.4	181.6	27.7	56	L	134.43	439	101,620	25,405	1.97
28	HBM676.SNVK.793	HBM687.KPKM.763	8242609fa	FFPE	44066	31299	W	M	91.4	181.6	27.7	56	L	136.11	586	55,200	13,800	3.55
29	HBM636.GVWP.354	HBM525.JNPV.685	cb2d976f4	FFPE	49548	34940	B	M	79.9	190.5	22	58	L	138.44	319	78,816	19,704	2.79
30	HBM725.PDDC.788	HBM525.JNPV.685	54f2eec69	FF	22240	30440	B	M	79.9	190.5	22	58	L	138.91	139	101,860	25,465	2.63
															**Average across all 30 slides**	80,498.47	20,124.62	

Note: Rows highlighted red are used in validation set.

Supplementary Table 4. HuBMAP colon metadata. This table provides metadata for the seven colon WSIs. Four of these dataset were sampled from a male donor and three from a female donor. For each WSI, we assigned a running number ID that is also used in **Fig. 2**. We provide corresponding HuBMAP sample and donor IDs, and the Kaggle IDs. We list race (White, W; Black or African American, B), sex (male, M; female, F), BMI, and age. We also computed the average crypt annotation area in square micrometers.

Supplementary Table 4. HuBMAP colon metadata

Slides	Sample Name	HuBMAP ID	Patient Number	Anatomical Structure	Race	Sex	BMI	Age	Average crypt area (pixels)	Average crypt area (µm^2)	Number of crypts
**31**	**HandE_B005_CL_b_RGB_bottomleft**	**HBM438.JXJW.249**	**B005**	**Transverse**	**W**	**F**	**23.24**	**24**	**18,428.90**	**13.90**	**36**
32	HandE_B005_CL_b_RGB_topleft	HBM353.NZVQ.793	B005	Descending	W	F	23.24	24	27,295.50	20.60	30
33	HandE_B005_CL_b_RGB_bottomright	HBM439.WJDV.974	B005	Sigmoid	W	F	23.24	24	13,674.90	10.30	37
34	CL_HandE_1234_topright	HBM938.KMNW.825	B004	Ascending	B	M	35.08	78	18,092.40	13.70	40
35	CL_HandE_1234_topleft	HBM334.QWFV.953	B004	Transverse	B	M	35.08	78	22,689.40	17.10	91
**36**	**CL_HandE_1234_bottomleft**	**HBM462.JKCN.863**	**B004**	**Descending**	**B**	**M**	**35.08**	**78**	**20,269.80**	**15.30**	**124**
37	CL_HandE_1234_bottomright	HBM575.THQM.284	B004	Sigmoid	B	M	35.08	78	41,300.10	31.20	35
								**Average across all 7 slides**	22,228.16	16.78	

Supplementary Table 5. HPA Kidney and colon metadata. This table provides metadata for the 157 kidney and colon WSIs in the HPA data. We list the age and sex of each WSI donor.

Supplementary Table 5. HPA kidney and colon metadata

ID	Organ	Sex	Age
45160_99047_A_7_5_kidney	kidney	M	59
42199_91531_A_7_5_kidney	kidney	M	59
39166_83439_A_9_5_kidney	kidney	F	41
45681_101950_A_7_5_kidney	kidney	M	70
36525_79274_A_8_5_kidney	kidney	M	28
64219_154500_A_9_5_kidney	kidney	M	73
44669_94899_A_7_5_kidney	kidney	M	70
44428_98682_A_7_5_kidney	kidney	F	68
48981_121052_A_9_5_kidney	kidney	M	61
41826_93039_A_7_5_kidney	kidney	F	68
52272_120106_A_9_5_kidney	kidney	M	61
54401_123069_A_9_5_kidney	kidney	M	61
52991_119723_A_9_5_kidney	kidney	M	61
41216_88604_A_7_5_kidney	kidney	M	59
48123_116109_A_7_5_kidney	kidney	F	68
43537_107119_A_7_5_kidney	kidney	M	70
38877_81110_A_7_5_kidney	kidney	M	70
42128_120967_A_9_5_kidney	kidney	M	61
43270_101867_A_9_5_kidney	kidney	F	41
37533_73616_A_8_5_kidney	kidney	M	28
76764_165426_A_9_5_kidney	kidney	M	73
75728_159183_A_9_5_kidney	kidney	M	73
36262_74534_A_8_5_kidney	kidney	M	28
36325_73367_A_8_5_kidney	kidney	M	28
63182_142050_A_9_5_kidney	kidney	M	73
35797_75610_A_8_5_kidney	kidney	M	28
64454_153783_A_9_5_kidney	kidney	M	73
36816_76605_A_9_5_kidney	kidney	F	56
34780_79927_A_9_5_kidney	kidney	F	41
37459_77928_A_9_5_kidney	kidney	F	56
39892_86708_A_9_5_kidney	kidney	F	41
44668_94884_A_7_5_kidney	kidney	M	70
36736_124947_A_9_5_kidney	kidney	M	61
46403_107840_A_9_5_kidney	kidney	F	56
36414_76912_A_9_5_kidney	kidney	F	56
53903_123092_A_9_5_kidney	kidney	M	61
36166_77504_A_9_5_kidney	kidney	F	56
35671_74699_A_8_5_kidney	kidney	M	28
35999_71854_A_7_5_kidney	kidney	M	70
66715_154628_A_9_5_kidney	kidney	M	73
38498_81651_A_7_5_kidney	kidney	F	68
37914_77828_A_7_5_kidney	kidney	F	68
51012_118065_A_8_5_kidney	kidney	M	59
64001_154953_A_8_5_kidney	kidney	F	41
40850_88383_A_7_5_kidney	kidney	M	70
37988_74968_A_9_5_kidney	kidney	F	56
41420_91575_A_9_5_kidney	kidney	F	41
75125_163802_A_9_5_kidney	kidney	M	73
36315_80549_A_7_5_kidney	kidney	F	68
44668_94884_A_9_5_kidney	kidney	F	56
48674_110605_A_8_5_kidney	kidney	M	28
48197_120913_A_9_5_kidney	kidney	M	61
35696_124478_A_9_5_kidney	kidney	M	61
49314_112479_A_7_5_kidney	kidney	F	68
44289_104479_A_7_5_kidney	kidney	M	70
53245_120116_A_8_5_kidney	kidney	F	41
45902_104617_A_7_5_kidney	kidney	M	70
49558_113972_A_7_5_kidney	kidney	M	70
52284_120401_A_9_5_kidney	kidney	M	61
47301_115898_A_7_5_kidney	kidney	M	70
52209_121910_A_9_5_kidney	kidney	M	61
73546_154496_A_9_5_kidney	kidney	M	73
37330_73560_A_8_5_kidney	kidney	M	28
35941_79509_A_9_5_kidney	kidney	F	56
51123_118600_A_8_5_kidney	kidney	M	59
39559_82979_A_8_5_kidney	kidney	M	28
41564_160015_A_9_5_kidney	kidney	M	73
45577_101774_A_9_5_kidney	kidney	F	56
47720_118410_A_8_5_kidney	kidney	M	59
71711_155943_A_9_5_kidney	kidney	M	73
41401_100305_A_8_5_kidney	kidney	M	28
50888_115749_A_7_5_kidney	kidney	F	68
37377_83024_A_7_5_kidney	kidney	F	68
45057_99501_A_9_5_kidney	kidney	F	41
36890_77656_A_9_5_kidney	kidney	F	56
52019_119795_A_8_5_kidney	kidney	F	41
38907_84055_A_7_5_kidney	kidney	M	70
50817_118074_A_8_5_kidney	kidney	M	59
45015_118657_A_7_5_kidney	kidney	F	68
44087_114637_A_7_5_kidney	kidney	M	59
44564_99172_A_9_5_kidney	kidney	F	41
44769_103022_A_7_5_kidney	kidney	F	68
61526_147227_A_9_5_kidney	kidney	M	73
36985_76861_A_9_5_kidney	kidney	F	56
51345_117779_A_8_5_kidney	kidney	M	59
37497_74170_A_7_5_kidney	kidney	M	70
51865_119729_A_9_5_kidney	kidney	M	61
35938_77229_A_7_5_kidney	kidney	M	59
43709_97584_A_7_5_kidney	kidney	F	68
36947_82934_A_8_5_kidney	kidney	M	28
34780_79927_A_8_5_kidney	kidney	M	28
49830_118215_A_8_5_kidney	kidney	M	59
41642_88213_A_7_5_kidney	kidney	M	59
51783_120541_A_9_5_kidney	kidney	M	61
65327_154791_A_8_5_kidney	kidney	F	41
36370_76786_A_7_5_kidney	kidney	M	59
68253_152489_A_8_5_kidney	kidney	F	41
48177_109920_A_9_5_kidney	kidney	F	56
66478_148787_A_8_5_kidney	kidney	F	41
50173_114448_A_9_3_largeintestine	colon	F	84
38723_81035_A_8_3_largeintestine	colon	F	79
69688_153740_A_8_3_largeintestine	colon	M	83
63633_149713_A_9_3_largeintestine	colon	F	84
72536_164996_A_8_3_largeintestine	colon	F	65
65958_157557_A_8_3_largeintestine	colon	M	83
51653_132579_A_8_3_largeintestine	colon	M	84
68212_152835_A_8_3_largeintestine	colon	M	83
77979_165557_A_8_3_largeintestine	colon	F	65
46057_118076_A_8_3_largeintestine	colon	M	84
39473_118918_A_8_3_largeintestine	colon	M	84
44005_102882_A_8_3_largeintestine	colon	F	79
68206_152278_A_8_3_largeintestine	colon	M	83
69409_153288_A_8_3_largeintestine	colon	M	83
46007_163049_A_8_3_largeintestine	colon	F	65
69907_159725_A_9_3_largeintestine	colon	F	84
39349_84104_A_8_3_largeintestine	colon	F	79
69439_153322_A_7_3_largeintestine	colon	F	65
44502_144146_A_8_3_largeintestine	colon	M	83
69409_153288_A_7_3_largeintestine	colon	F	65
68244_152760_A_8_3_largeintestine	colon	M	83
39255_84732_A_8_3_largeintestine	colon	F	79
36407_76610_A_8_3_largeintestine	colon	F	79
64872_149526_A_8_3_largeintestine	colon	M	83
67685_155267_A_9_3_largeintestine	colon	F	84
38013_80093_A_8_3_largeintestine	colon	F	79
47333_105872_A_9_3_largeintestine	colon	F	84
50977_121025_A_9_3_largeintestine	colon	F	84
43141_140599_A_8_3_largeintestine	colon	M	83
56768_152680_A_8_3_largeintestine	colon	M	83
64095_156888_A_8_3_largeintestine	colon	M	83
45244_103480_A_8_3_largeintestine	colon	M	84
55969_127605_A_8_3_largeintestine	colon	M	84
72629_158408_A_8_3_largeintestine	colon	M	83
42844_98171_A_8_3_largeintestine	colon	M	84
45951_103533_A_8_3_largeintestine	colon	M	84
38034_80453_A_8_3_largeintestine	colon	F	79
35275_160325_A_8_3_largeintestine	colon	F	65
48368_117291_A_9_3_largeintestine	colon	F	84
60227_134089_A_8_3_largeintestine	colon	M	84
57585_163806_A_8_3_largeintestine	colon	F	65
43499_166208_A_8_3_largeintestine	colon	F	65
42268_116797_A_8_3_largeintestine	colon	M	84
73705_161912_A_8_3_largeintestine	colon	F	65
49979_115386_A_8_3_largeintestine	colon	M	84
48992_118293_A_8_3_largeintestine	colon	M	84
61503_143161_A_9_3_largeintestine	colon	F	84
64946_144077_A_8_3_largeintestine	colon	M	83
52398_123232_A_9_3_largeintestine	colon	F	84
71287_154618_A_9_3_largeintestine	colon	F	84
56230_133242_A_9_3_largeintestine	colon	F	84
51564_117501_A_8_3_largeintestine	colon	F	55
77757_165553_A_8_3_largeintestine	colon	F	65
46992_117883_A_8_3_largeintestine	colon	M	84
41089_87420_A_8_3_largeintestine	colon	F	79
68239_150613_A_7_3_largeintestine	colon	M	47
57980_145448_A_9_3_largeintestine	colon	F	84
55846_137737_A_9_3_largeintestine	colon	F	84

Supplementary Table 6. Algorithm performance on HuBMAP data without watershed. This table lists Dice coefficients, false negatives (FN), true positives (TP), and false positives (FP) of winning algorithms for individual WSIs in all three predicted datasets (10 kidney WSIs using Strategy 1, 2 colon WSIs using Strategy 2, 2 colon WSIs using Strategy 3. Threshold used for calculations is 0.5.

Supplementary Table 6. Algorithm performance on HuBMAP data without watershed

Kaggle reproduced (kidney)

		Ground truth	Tom				Gleb				Whats goin on				DeepLive.exe				Deepflash2				DICE accross 5 models
Slide	HuBMAP ID	#FTUs	DICE	FN	TP	FP	DICE	FN	TP	FP	DICE	FN	TP	FP	DICE	FN	TP	FP	DICE	FN	TP	FP	DICE
4	HBM324.ZGZM.874	315	0.966	1	314	4	0.969	2	313	4	0.969	1	314	4	0.966	1	314	6	0.961	0	315	9	0.966
5	HBM649.DLZF.463	157	0.953	3	154	3	0.951	5	152	5	0.953	3	154	6	0.954	3	154	5	0.953	2	155	8	0.953
6	HBM296.RLWW.755	355	0.968	2	353	2	0.968	4	351	6	0.968	3	352	7	0.967	1	354	5	0.963	1	354	7	0.967
8	HBM649.XFQG.775	341	0.966	7	334	4	0.969	7	334	4	0.967	7	334	5	0.966	7	334	7	0.963	2	339	8	0.966
13	HBM673.JJRZ.435	51	0.934	2	49	2	0.929	2	49	2	0.927	1	50	2	0.924	1	50	4	0.924	0	51	4	0.928
16	HBM344.LLLV.539	320	0.933	9	311	14	0.927	16	304	31	0.927	16	304	18	0.933	7	313	23	0.929	11	309	21	0.930
17	HBM627.RSGW.898	97	0.943	2	95	5	0.952	1	96	4	0.953	2	95	4	0.947	2	95	5	0.941	1	96	8	0.947
18	HBM227.THVC.544	109	0.961	1	108	2	0.959	4	105	3	0.958	3	106	2	0.961	1	108	3	0.957	1	108	3	0.959
23	HBM832.FQKR.463	118	0.934	4	114	5	0.932	4	114	5	0.935	2	116	7	0.937	1	117	7	0.931	5	113	5	0.934
26	HBM984.PMZN.942	175	0.957	5	170	2	0.944	10	165	5	0.946	9	166	3	0.948	6	169	7	0.953	4	171	8	0.949

Transfer learning (trained on kidney & colon, tested on colon)

		Ground truth	Tom				Gleb				Whats goin on				DeepLive.exe				Deepflash2				DICE accross 5 models
Slide	HuBMAP ID	#FTUs	DICE	FN	TP	FP	DICE	FN	TP	FP	DICE	FN	TP	FP	DICE	FN	TP	FP	DICE	FN	TP	FP	DICE
31	HBM438.JXJW.249	36	0.832	2	34	12	0.764	10	26	8	0.747	2	34	16	0.823	2	34	10	0.653	11	25	29	0.764
36	HBM462.JKCN.863	124	0.928	7	117	5	0.912	10	114	2	0.888	5	119	11	0.931	6	118	9	0.780	25	99	46	0.888

From scratch (trained on colon, tested on colon)

		Ground truth	Tom				Gleb				Whats goin on				DeepLive.exe				Deepflash2				DICE accross 5 models
Slide	HuBMAP ID	#FTUs	DICE	FN	TP	FP	DICE	FN	TP	FP	DICE	FN	TP	FP	DICE	FN	TP	FP	DICE	FN	TP	FP	DICE
31	HBM438.JXJW.249	36	0.823	1	35	15	0.748	10	26	7	0.707	3	33	18	0.850	1	35	12	0.747	4	32	21	0.775
36	HBM462.JKCN.863	124	0.928	6	118	10	0.902	15	109	6	0.875	3	121	11	0.936	6	118	3	0.865	9	115	11	0.901

Supplementary Table 7. Algorithm performance on HuBMAP data with watershed. This table lists Dice coefficients, false negatives (FN), true positives (TP), and false positives (FP) of winning algorithms for individual WSIs in all three predicted datasets (10 kidney WSIs using strategy 1, 2 colon WSIs using Strategy 2, 2 colon WSIs using Strategy 3. Threshold used for calculations is 0.5. For watershed, a minimum distance of 30 for kidney and 60 for colon data is used.

Supplementary Table 7. Algorithm performance on HuBMAP data with watershed

Kaggle reproduced (kidney)																							
		Ground truth	Tom				Gleb				Whats goin on				DeepLive.exe				Deepflash2				DICE accross 5 models
Slide	HuBMAP ID	#FTUs	DICE	FN	TP	FP	DICE	FN	TP	FP	DICE	FN	TP	FP	DICE	FN	TP	FP	DICE	FN	TP	FP	DICE
4	HBM324.ZGZM.874	315	0.966	10	305	4	0.969	7	308	4	0.969	9	306	4	0.966	5	310	6	0.961	9	306	9	0.966
5	HBM649.DLZF.463	157	0.953	7	150	2	0.951	7	150	5	0.953	6	151	5	0.954	3	154	5	0.953	6	151	8	0.953
6	HBM296.RLWW.755	355	0.968	12	343	2	0.968	7	348	6	0.968	9	346	7	0.967	6	349	5	0.963	13	342	8	0.967
8	HBM649.XFQG.775	341	0.966	19	322	4	0.969	12	329	4	0.967	17	324	5	0.966	16	325	6	0.963	14	327	8	0.966
13	HBM673.JJRZ.435	51	0.934	3	48	2	0.929	3	48	2	0.927	2	49	2	0.924	2	49	4	0.924	1	50	3	0.928
16	HBM344.LLLV.539	320	0.933	23	297	13	0.927	22	298	21	0.927	27	293	16	0.933	16	304	22	0.929	22	298	21	0.930
17	HBM627.RSGW.898	97	0.943	3	94	5	0.952	2	95	4	0.953	2	95	4	0.947	2	95	5	0.941	1	96	8	0.947
18	HBM227.THVC.544	109	0.961	1	108	2	0.959	4	105	3	0.958	3	106	2	0.961	1	108	3	0.957	1	108	3	0.959
23	HBM832.FQKR.463	118	0.934	8	110	5	0.932	6	112	5	0.935	7	111	7	0.937	4	114	7	0.931	8	110	5	0.934
26	HBM984.PMZN.942	175	0.957	9	166	2	0.944	13	162	6	0.946	13	162	3	0.948	8	167	7	0.953	8	167	7	0.949
Transfer learning (trained on kidney & colon, tested on colon)																							
		Ground truth	Tom				Gleb				Whats goin on				DeepLive.exe				Deepflash2				DICE accross 5 models
Slide	HuBMAP ID	#FTUs	DICE	FN	TP	FP	DICE	FN	TP	FP	DICE	FN	TP	FP	DICE	FN	TP	FP	DICE	FN	TP	FP	DICE
31	HBM438.JXJW.249	36	0.832	5	31	9	0.764	15	21	5	0.747	17	19	14	0.823	3	33	9	0.653	8	28	17	0.764
36	HBM462.JKCN.863	124	0.928	47	77	5	0.912	61	63	5	0.888	101	23	18	0.931	23	101	2	0.780	25	99	10	0.888
From scratch (trained on colon, tested on colon)																							
		Ground truth	Tom				Gleb				Whats goin on				DeepLive.exe				Deepflash2				DICE accross 5 models
Slide	HuBMAP ID	#FTUs	DICE	FN	TP	FP	DICE	FN	TP	FP	DICE	FN	TP	FP	DICE	FN	TP	FP	DICE	FN	TP	FP	DICE
31	HBM438.JXJW.249	36	0.823	5	31	9	0.748	15	21	5	0.707	17	19	14	0.850	3	33	9	0.747	8	28	17	0.775
36	HBM462.JKCN.863	124	0.928	47	77	5	0.902	61	63	5	0.875	101	23	18	0.936	23	101	2	0.865	25	99	10	0.901

Supplementary Table 8. Algorithm performance on kidney HPA data. This table lists Dice coefficients, false negatives (FN), true positives (TP), and false positives (FP) of winning algorithms for individual WSIs in kidney HPA data (using Strategy 4). Threshold used for calculations is 0.5. For watershed, a minimum distance of 80 is used.

Supplementary Table 8. Algorithm performance on kidney HPA data

		HPA Kidney																			
	Ground Truth	Tom				Gleb				Whats Goin On				Deeplive.exe				Deepflash2			
ID	#FTUs	Dice	FP	TP	FN	Dice	FP	TP	FN	Dice	FP	TP	FN	Dice	FP	TP	FN	Dice	FP	TP	FN
45160_99047_A_7_5_kidney	1	0.08	2	0	1	0	0	0	0	0	0	0	0	0	0	0	0	0	57	0	1
42199_91531_A_7_5_kidney	3	0.98	0	3	0	0.99	0	3	0	0.97	0	3	0	0.99	0	3	0	0.05	56	0	3
39166_83439_A_9_5_kidney	3	0.99	0	3	0	0.99	0	3	0	0.95	0	3	0	0.98	0	3	0	0.08	48	0	3
45681_101950_A_7_5_kidney	7	0.87	0	4	3	0.76	1	3	4	0.07	1	0	7	0.75	1	3	4	0.01	60	0	7
36525_79274_A_8_5_kidney	3	0.97	0	3	0	0.86	1	2	1	0.54	1	1	2	0.81	0	2	1	0.11	66	0	3
64219_154500_A_9_5_kidney	3	0.9	1	2	1	0.84	0	2	1	0.82	0	2	1	0.84	0	2	1	0.03	73	0	3
44669_94899_A_7_5_kidney	1	0.97	0	1	0	0.98	0	1	0	0.96	0	1	0	0.97	0	1	0	0	69	0	1
44428_98682_A_7_5_kidney	2	0.96	1	2	0	0.99	0	2	0	0.99	0	2	0	0.99	0	2	0	0.1	60	1	1
48981_121052_A_9_5_kidney	6	0.95	0	5	1	0.83	0	4	2	0.81	1	4	2	0.95	0	5	1	0.08	71	1	5
41826_93039_A_7_5_kidney	4	0.98	0	4	0	0.99	0	4	0	0.92	0	3	1	0.96	1	3	1	0.06	47	1	3
52272_120106_A_9_5_kidney	4	0.62	0	2	2	0	0	0	0	0.1	1	0	4	0.63	0	2	2	0.08	57	1	3
54401_123069_A_9_5_kidney	2	0.98	0	2	0	0.98	0	2	0	0.95	0	2	0	0.97	0	2	0	0.29	49	2	0
52991_119723_A_9_5_kidney	5	0.62	0	2	3	0	0	0	0	0	0	0	0	0.27	3	0	5	0.06	71	1	4
41216_88604_A_7_5_kidney	7	0.92	0	6	1	0.95	0	6	1	0.69	0	3	4	0.94	0	6	1	0.12	61	2	5
48123_116109_A_7_5_kidney	7	0.96	0	7	0	0.97	0	7	0	0.46	1	2	5	0.95	0	7	0	0.24	61	4	3
43537_107119_A_7_5_kidney	5	0.98	0	5	0	0.98	0	4	1	0.36	0	2	3	0.98	0	5	0	0.1	71	0	5
38877_81110_A_7_5_kidney	4	0.99	0	4	0	0.99	0	4	0	0	0	0	0	0.96	0	4	0	0.14	71	2	2
42128_120967_A_9_5_kidney	3	0.98	0	3	0	0.98	0	3	0	0	0	0	0	0.97	0	3	0	0.12	43	0	3
43270_101867_A_9_5_kidney	1	0.98	0	1	0	0.99	0	1	0	0.91	0	1	0	0.98	0	1	0	0.04	48	1	0
37533_73616_A_8_5_kidney	4	0.69	0	2	2	0.69	0	2	2	0	0	0	0	0.05	1	0	4	0	0	0	0
76764_165426_A_9_5_kidney	3	0.65	1	1	2	0.89	0	2	1	0.13	1	0	3	0.94	0	3	0	0.08	77	0	3
75728_159183_A_9_5_kidney	3	0.97	0	3	0	0.99	0	3	0	0	0	0	0	0.97	0	3	0	0.06	46	0	3
36262_74534_A_8_5_kidney	6	0.52	5	2	4	0.38	0	1	5	0.18	0	1	5	0.42	1	1	5	0.01	54	0	6
36325_73367_A_8_5_kidney	1	0.2	7	0	1	0	0	0	0	0	0	0	0	0	0	0	0	0.01	58	0	1
63182_142050_A_9_5_kidney	1	0.98	0	1	0	0.94	0	1	0	0	0	0	0	0.97	0	1	0	0.02	81	0	1
35797_75610_A_8_5_kidney	4	0.74	0	2	2	0.72	0	2	2	0	0	0	0	0	0	0	0	0	64	0	4
64454_153783_A_9_5_kidney	3	0.95	0	3	0	0.89	0	2	1	0.69	0	1	2	0.88	0	2	1	0.04	84	0	3
36816_76605_A_9_5_kidney	4	0.66	4	2	2	0.61	0	2	2	0	0	0	0	0.1	1	0	4	0.01	71	0	4
34780_79927_A_9_5_kidney	4	0.84	0	2	2	0.85	0	2	2	0.11	2	0	4	0.84	0	2	2	0	49	0	4
37459_77928_A_9_5_kidney	5	0.65	2	2	3	0.66	0	2	3	0	0	0	0	0.07	4	0	5	0.01	58	0	5
39892_86708_A_9_5_kidney	4	0.97	0	4	0	0.98	0	4	0	0.84	0	3	1	0.97	0	4	0	0.13	55	1	3
44668_94884_A_7_5_kidney	1	0.69	4	1	0	0.96	0	1	0	0.73	0	1	0	0.97	0	1	0	0	48	0	1
36736_124947_A_9_5_kidney	2	0.98	0	2	0	0.99	0	2	0	0.95	0	2	0	0.97	0	2	0	0.02	58	0	2
46403_107840_A_9_5_kidney	4	0.95	0	4	0	0.99	0	4	0	0.93	0	4	0	0.99	0	4	0	0.03	46	0	4
36414_76912_A_9_5_kidney	2	0.97	0	2	0	0.99	0	2	0	0	0	0	0	0.97	0	2	0	0.02	59	0	2
53903_123092_A_9_5_kidney	5	0.9	0	4	1	0.43	0	2	3	0.08	1	0	5	0.65	0	2	3	0.05	52	0	5
36166_77504_A_9_5_kidney	4	0.87	0	4	0	0.81	0	3	1	0	0	0	0	0.8	0	3	1	0.04	52	0	4
35671_74699_A_8_5_kidney	2	0.61	0	1	1	0.66	0	1	1	0	0	0	0	0	0	0	0	0	46	0	2
35999_71854_A_7_5_kidney	3	0.52	5	2	1	0	0	0	0	0	0	0	0	0.05	1	0	3	0	62	0	3
66715_154628_A_9_5_kidney	2	0.93	2	2	0	0.98	0	2	0	0.8	0	2	0	0.98	0	2	0	0.05	69	0	2
38498_81651_A_7_5_kidney	7	0.97	0	7	0	0.8	0	5	2	0.67	0	4	3	0.94	2	6	1	0.16	64	1	6
37914_77828_A_7_5_kidney	4	0.6	0	3	1	0.2	0	1	3	0.19	0	1	3	0.58	2	1	3	0.05	45	1	3
51012_118065_A_8_5_kidney	4	0.92	1	3	1	0.96	0	3	1	0.95	0	3	1	0.93	0	3	1	0.2	63	3	1
64001_154953_A_8_5_kidney	4	0.9	1	3	1	0.97	0	4	0	0.82	0	3	1	0.97	0	4	0	0.29	59	2	2
40850_88383_A_7_5_kidney	4	0.98	0	4	0	0.98	0	4	0	0.35	0	1	3	0.94	0	4	0	0.17	51	2	2
37988_74968_A_9_5_kidney	3	0.92	0	3	0	0.98	0	3	0	0	0	0	0	0.4	1	1	2	0.05	60	0	3
41420_91575_A_9_5_kidney	2	0.99	0	2	0	0.99	0	2	0	0.57	0	1	1	0.96	0	2	0	0.1	42	1	1
75125_163802_A_9_5_kidney	2	0.9	2	2	0	0.61	2	1	1	0	0	0	0	0.84	0	2	0	0.02	67	0	2
36315_80549_A_7_5_kidney	4	0.88	0	3	1	0.94	0	3	1	0.58	0	1	3	0.79	1	2	2	0.11	65	0	4
44668_94884_A_9_5_kidney	2	0.79	0	1	1	0.79	0	1	1	0	0	0	0	0.78	0	1	1	0	64	0	2
48674_110605_A_8_5_kidney	3	0.98	0	3	0	0.98	0	3	0	0	0	0	0	0.96	0	3	0	0.04	61	0	3
48197_120913_A_9_5_kidney	4	0.64	1	2	2	0	0	0	0	0.42	0	1	3	0.72	0	2	2	0.05	71	0	4
35696_124478_A_9_5_kidney	2	0.83	1	1	1	0.06	1	0	2	0.65	0	1	1	0.71	0	1	1	0.02	84	0	2
49314_112479_A_7_5_kidney	4	0.92	0	3	1	0.95	0	3	1	0.35	0	1	3	0.9	0	3	1	0.05	40	1	3
44289_104479_A_7_5_kidney	7	0.95	0	6	1	0.9	0	5	2	0.8	2	3	4	0.95	0	6	1	0.17	75	0	7
53245_120116_A_8_5_kidney	3	0.91	0	3	0	0.77	0	2	1	0.77	0	2	1	0.84	1	2	1	0.12	49	1	2
45902_104617_A_7_5_kidney	2	0.98	0	2	0	0.99	0	2	0	0.74	0	1	1	0.75	0	1	1	0.1	83	2	0
49558_113972_A_7_5_kidney	4	0.98	0	4	0	0.99	0	4	0	0.73	1	3	1	0.86	1	3	1	0.11	61	0	4
52284_120401_A_9_5_kidney	2	0.98	0	2	0	0.99	0	2	0	0.73	0	1	1	0.97	0	2	0	0.08	52	1	1
47301_115898_A_7_5_kidney	4	0.83	0	3	1	0.84	0	3	1	0.44	0	2	2	0.82	0	3	1	0.14	69	1	3
52209_121910_A_9_5_kidney	3	0.97	0	3	0	0.97	0	3	0	0.42	1	1	2	0.93	0	3	0	0.1	51	1	2
73546_154496_A_9_5_kidney	3	0.98	0	3	0	0.86	0	2	1	0.02	1	0	3	0.48	1	1	2	0.05	73	1	2
37330_73560_A_8_5_kidney	2	0.95	0	2	0	0.65	0	1	1	0	0	0	0	0.63	0	1	1	0.01	68	0	2
35941_79509_A_9_5_kidney	3	0.45	6	1	2	0.61	0	1	2	0	0	0	0	0.86	0	2	1	0.04	66	0	3
51123_118600_A_8_5_kidney	2	0.87	0	2	0	0.7	0	1	1	0.68	0	1	1	0.97	0	2	0	0.1	63	1	1
39559_82979_A_8_5_kidney	6	0.52	1	2	4	0	0	0	0	0	0	0	0	0	0	0	0	0.04	47	0	6
41564_160015_A_9_5_kidney	3	0.96	0	3	0	0	0	0	0	0	0	0	0	0.82	0	3	0	0.04	75	1	2
45577_101774_A_9_5_kidney	2	0.78	0	1	1	0.79	0	1	1	0	0	0	0	0.78	0	1	1	0.1	56	1	1
47720_118410_A_8_5_kidney	2	0.98	0	2	0	0.98	0	2	0	0.95	0	2	0	0.96	0	2	0	0.06	61	1	1
71711_155943_A_9_5_kidney	3	0.76	0	2	1	0	0	0	0	0	0	0	0	0.73	0	2	1	0.01	62	0	3
41401_100305_A_8_5_kidney	2	0.3	1	1	1	0	0	0	0	0	0	0	0	0.2	1	0	2	0.03	56	1	1
50888_115749_A_7_5_kidney	1	0.75	1	1	0	0.97	0	1	0	0	0	0	0	0.93	0	1	0	0.07	56	1	0
37377_83024_A_7_5_kidney	2	0.99	0	2	0	0.99	0	2	0	0	0	0	0	0.95	0	2	0	0.14	66	2	0
45057_99501_A_9_5_kidney	4	0.98	0	4	0	0.99	0	4	0	0.55	1	1	3	0.98	0	4	0	0.22	68	1	3
36890_77656_A_9_5_kidney	6	0.85	0	5	1	0.94	0	5	1	0.55	0	2	4	0.86	0	4	2	0.01	65	0	6
52019_119795_A_8_5_kidney	6	0.92	0	5	1	0.92	0	5	1	0.79	0	4	2	0.92	0	5	1	0.2	74	2	4
38907_84055_A_7_5_kidney	4	0.8	1	2	2	0.8	1	2	2	0.71	0	1	3	0.8	0	3	1	0.04	81	0	4
50817_118074_A_8_5_kidney	2	0.73	1	1	1	0.79	0	1	1	0.48	0	1	1	0.78	0	1	1	0.06	78	1	1
45015_118657_A_7_5_kidney	2	0.99	0	2	0	0.99	0	2	0	0.98	0	2	0	0.99	0	2	0	0.05	64	0	2
44087_114637_A_7_5_kidney	1	0.74	0	1	0	0	0	0	0	0	0	0	0	0	0	0	0	0	62	0	1
44564_99172_A_9_5_kidney	6	0.86	0	4	2	0	0	0	0	0	0	0	0	0.78	0	3	3	0.07	45	1	5
44769_103022_A_7_5_kidney	2	0.99	0	2	0	0.96	0	2	0	0.49	2	1	1	0.96	0	2	0	0.07	48	1	1
61526_147227_A_9_5_kidney	2	0.96	0	2	0	0.83	2	1	1	0	0	0	0	0.78	0	1	1	0.08	66	1	1
36985_76861_A_9_5_kidney	2	0.95	0	2	0	0.96	0	2	0	0	0	0	0	0.94	0	2	0	0.08	57	1	1
51345_117779_A_8_5_kidney	5	0.97	0	5	0	0.97	0	5	0	0.86	0	4	1	0.89	0	4	1	0.13	88	1	4
37497_74170_A_7_5_kidney	3	0.73	4	3	0	0.8	0	2	1	0	0	0	0	0.97	0	3	0	0.07	77	0	3
51865_119729_A_9_5_kidney	4	0.88	0	3	1	0.89	0	3	1	0.8	1	2	2	0.9	0	3	1	0.04	58	0	4
35938_77229_A_7_5_kidney	3	0.95	1	3	0	0.99	0	3	0	0.95	0	3	0	0.98	0	3	0	0.1	76	1	2
43709_97584_A_7_5_kidney	7	0.92	0	5	2	0.92	0	5	2	0.75	1	4	3	0.91	0	5	2	0.08	72	1	6
36947_82934_A_8_5_kidney	3	0.69	0	2	1	0	0	0	0	0	0	0	0	0.43	1	1	2	0.03	63	0	3
34780_79927_A_8_5_kidney	6	0.35	1	2	4	0	0	0	0	0	0	0	0	0.02	1	0	6	0	48	0	6
49830_118215_A_8_5_kidney	1	0	0	0	0	0	0	0	0	0	0	0	0	0	0	0	0	0	75	0	1
41642_88213_A_7_5_kidney	4	0.94	0	4	0	0.97	0	4	0	0.36	0	1	3	0.95	0	4	0	0.02	75	0	4
51783_120541_A_9_5_kidney	2	0.98	0	2	0	0.99	0	2	0	0.96	0	2	0	0.97	0	2	0	0.11	41	1	1
65327_154791_A_8_5_kidney	7	0.84	0	5	2	0.61	5	4	3	0.09	3	0	7	0.75	0	4	3	0.26	70	4	3
36370_76786_A_7_5_kidney	2	0.88	0	1	1	0.89	0	1	1	0	0	0	0	0	0	0	0	0.03	52	0	2
68253_152489_A_8_5_kidney	6	0.96	0	5	1	0.97	0	5	1	0.88	0	4	2	0.95	0	5	1	0.21	52	1	5
48177_109920_A_9_5_kidney	2	0.75	1	2	0	0.97	0	2	0	0	0	0	0	0.9	0	2	0	0.19	49	0	2
66478_148787_A_8_5_kidney	2	0.65	0	1	1	0.66	0	1	1	0.03	0	0	0	0.64	0	1	1	0.05	50	1	1
**Mean Dice**		0.83				0.73				0.38				0.74				0.07			
**Total GT, FP, TP, FN**	337		58	263	73		13	217	71		22	108	119		25	222	97		6007	61	272
**Precision**		0.82				0.94				0.83				0.90				0.01			
**Recall**		0.78				0.75				0.48				0.70				0.18			

Supplementary Table 9. Algorithm performance on colon HPA data. This table lists Dice coefficients, false negatives (FN), true positives (TP), and false positives (FP) of winning algorithms for individual WSIs in colon HPA data (using Strategy 5). Threshold used for calculations is 0.5. For watershed, a minimum distance of 80 is used.

Supplementary Table 9. Algorithm performance on colon HPA data

	HPA Colon																				
	Ground Truth	Tom				Gleb				Whats Goin On				Deeplive.exe				Deepflash2			
ID	#FTUs	Dice	FP	TP	FN	Dice	FP	TP	FN	Dice	FP	TP	FN	Dice	FP	TP	FN	Dice	FP	TP	FN
50173_114448_A_9_3_largeintestine	106	0.2	14	5	101	0.75	5	33	73	0.42	29	33	73	0.69	4	34	72	0.57	11	40	66
38723_81035_A_8_3_largeintestine	34	0	1	0	34	0.06	2	1	33	0.32	27	4	30	0.05	4	1	33	0.1	7	1	33
69688_153740_A_8_3_largeintestine	36	0.09	3	7	29	0.82	1	28	8	0.47	20	21	15	0.61	4	25	11	0.53	28	17	19
63633_149713_A_9_3_largeintestine	54	0.35	11	16	38	0.72	2	23	31	0.57	22	26	28	0.62	7	16	38	0.23	23	8	46
72536_164996_A_8_3_largeintestine	72	0.57	16	26	46	0.77	6	30	42	0.58	21	23	49	0.76	9	27	45	0.05	6	1	71
65958_157557_A_8_3_largeintestine	41	0.11	10	2	39	0.83	0	34	7	0.44	30	17	24	0.46	6	16	25	0.32	27	8	33
51653_132579_A_8_3_largeintestine	41	0.01	1	1	40	0.57	2	18	23	0.25	34	5	36	0.2	15	4	37	0.04	12	0	41
68212_152835_A_8_3_largeintestine	38	0.02	4	0	38	0.61	4	19	19	0.22	25	3	35	0.01	2	0	38	0.22	25	6	32
77979_165557_A_8_3_largeintestine	31	0.21	7	6	25	0.74	6	15	16	0.59	27	13	18	0.76	8	14	17	0.04	12	0	31
46057_118076_A_8_3_largeintestine	57	0.02	4	0	57	0.59	8	20	37	0.26	26	13	44	0.36	20	17	40	0.13	18	6	51
39473_118918_A_8_3_largeintestine	86	0.11	13	4	82	0.47	6	21	65	0.48	30	37	49	0.18	7	7	79	0.2	24	9	77
44005_102882_A_8_3_largeintestine	87	0.06	4	5	82	0.34	5	13	74	0.35	30	24	63	0.09	9	4	83	0.07	16	3	84
68206_152278_A_8_3_largeintestine	38	0	0	0	0	0.67	3	19	19	0.17	23	3	35	0.22	17	5	33	0.21	27	2	36
69409_153288_A_8_3_largeintestine	45	0.13	10	3	42	0.76	1	28	17	0.46	26	20	25	0.32	12	9	36	0.2	27	1	44
46007_163049_A_8_3_largeintestine	47	0.48	17	13	34	0.66	1	17	30	0.45	16	17	30	0.68	7	12	35	0.01	25	0	47
69907_159725_A_9_3_largeintestine	62	0.21	10	9	51	0.77	4	36	24	0.36	27	19	41	0.65	6	28	32	0.34	14	12	48
39349_84104_A_8_3_largeintestine	37	0.09	2	5	32	0.62	1	13	24	0.43	24	11	26	0.35	5	6	31	0.18	16	3	34
69439_153322_A_7_3_largeintestine	67	0.56	31	21	46	0.76	4	22	45	0.55	22	30	37	0.6	12	13	54	0.01	2	0	67
44502_144146_A_8_3_largeintestine	48	0.1	15	1	47	0.83	0	34	14	0.61	15	35	13	0.56	11	21	27	0.6	43	20	28
69409_153288_A_7_3_largeintestine	70	0.56	25	21	49	0.79	2	26	44	0.58	25	27	43	0.69	9	16	54	0.01	3	0	70
68244_152760_A_8_3_largeintestine	33	0.03	1	1	32	0.55	1	13	20	0.4	18	15	18	0.01	2	0	33	0.36	32	11	22
39255_84732_A_8_3_largeintestine	70	0.06	6	0	70	0.65	3	19	51	0.34	30	20	50	0.41	15	16	54	0.29	29	11	59
36407_76610_A_8_3_largeintestine	44	0.13	9	4	40	0.71	5	27	17	0.42	27	18	26	0.59	3	18	26	0.41	18	15	29
64872_149526_A_8_3_largeintestine	34	0.01	1	0	34	0.84	2	21	13	0.55	13	17	17	0.57	2	11	23	0.62	19	20	14
67685_155267_A_9_3_largeintestine	41	0	0	0	0	0.52	2	16	24	0.25	25	8	32	0.42	3	16	24	0.28	19	9	31
38013_80093_A_8_3_largeintestine	71	0.11	4	7	64	0.35	12	10	61	0.33	34	20	51	0.39	18	16	55	0.07	18	1	70
47333_105872_A_9_3_largeintestine	154	0.71	17	79	75	0.92	4	102	52	0.69	22	124	30	0.9	8	93	61	0.74	21	100	54
50977_121025_A_9_3_largeintestine	79	0.24	9	16	63	0.75	2	34	45	0.41	26	33	46	0.46	5	19	60	0.27	25	17	62
43141_140599_A_8_3_largeintestine	88	0.22	24	14	74	0.86	0	55	33	0.58	21	63	25	0.4	16	29	59	0.76	48	19	69
56768_152680_A_8_3_largeintestine	13	0	0	0	0	0.7	1	7	6	0.25	13	0	13	0.39	3	4	9	0.49	17	6	7
64095_156888_A_8_3_largeintestine	34	0.01	1	0	34	0.71	1	25	9	0.33	27	6	28	0.12	5	3	31	0.39	33	7	27
45244_103480_A_8_3_largeintestine	109	0.27	24	21	88	0.63	4	54	55	0.45	18	62	47	0.22	10	17	92	0.18	29	9	100
55969_127605_A_8_3_largeintestine	67	0.55	16	30	35	0.91	2	35	30	0.74	7	50	15	0.84	5	25	40	0.3	18	13	52
72629_158408_A_8_3_largeintestine	45	0.1	9	2	43	0.76	0	29	16	0.42	19	21	24	0.07	2	2	43	0.15	15	2	43
42844_98171_A_8_3_largeintestine	31	0.11	14	2	29	0.68	9	17	14	0.27	20	5	26	0.74	4	16	15	0.08	24	2	29
45951_103533_A_8_3_largeintestine	118	0.26	20	22	96	0.73	6	65	53	0.57	24	75	43	0.34	6	33	85	0.33	38	25	93
38034_80453_A_8_3_largeintestine	35	0.14	5	2	33	0.68	0	17	18	0.53	15	17	18	0.38	4	7	28	0.24	16	7	28
35275_160325_A_8_3_largeintestine	31	0.08	6	1	30	0.83	2	12	19	0.45	37	6	25	0.82	3	14	17	0	17	0	31
48368_117291_A_9_3_largeintestine	43	0.05	4	2	41	0.53	2	15	28	0.17	19	4	39	0.31	6	8	35	0.21	16	7	36
60227_134089_A_8_3_largeintestine	54	0.31	9	16	38	0.73	2	30	24	0.55	10	36	18	0.49	5	16	38	0.31	13	8	46
57585_163806_A_8_3_largeintestine	26	0.42	9	8	18	0.89	1	21	5	0.74	18	19	7	0.88	0	19	7	0.01	12	0	26
43499_166208_A_8_3_largeintestine	53	0.45	6	13	40	0.96	4	15	38	0.8	5	33	20	0.95	5	16	37	0.03	7	1	52
42268_116797_A_8_3_largeintestine	48	0.02	2	1	47	0.55	5	19	29	0.2	38	1	47	0.34	12	9	39	0.05	6	1	47
73705_161912_A_8_3_largeintestine	13	0.23	13	2	11	0.84	1	8	5	0.58	18	9	4	0.82	2	8	5	0.02	25	0	13
49979_115386_A_8_3_largeintestine	47	0.09	8	1	46	0.69	5	17	30	0.3	24	9	38	0.59	3	16	31	0.02	4	0	47
48992_118293_A_8_3_largeintestine	50	0.14	23	3	47	0.55	6	25	25	0.29	38	7	43	0.36	10	16	34	0.09	10	3	47
61503_143161_A_9_3_largeintestine	56	0.62	7	30	26	0.74	3	27	29	0.68	7	35	21	0.73	6	17	39	0.67	21	11	45
64946_144077_A_8_3_largeintestine	35	0.02	3	0	35	0.57	6	18	17	0.4	24	13	22	0.08	5	1	34	0.13	25	0	35
52398_123232_A_9_3_largeintestine	19	0.01	7	0	19	0.72	1	10	9	0.41	14	4	15	0.61	1	9	10	0.08	14	1	18
71287_154618_A_9_3_largeintestine	93	0.72	13	54	38	0.88	4	52	40	0.72	11	67	25	0.83	8	37	55	0.5	26	41	51
56230_133242_A_9_3_largeintestine	91	0.39	8	26	65	0.7	3	27	64	0.44	33	36	55	0.6	9	30	61	0.65	17	18	73
51564_117501_A_8_3_largeintestine	4	0.16	1	1	3	0.22	0	1	3	0.18	3	2	2	0.18	0	1	3	0.12	28	1	3
77757_165553_A_8_3_largeintestine	34	0.41	9	7	27	0.87	1	13	21	0.74	16	14	20	0.84	1	15	19	0.02	3	1	33
46992_117883_A_8_3_largeintestine	51	0.16	7	6	45	0.65	3	20	31	0.27	31	5	46	0.36	7	9	42	0.23	9	8	43
41089_87420_A_8_3_largeintestine	48	0	2	0	48	0.52	5	12	36	0.11	20	1	47	0.3	12	9	39	0.17	31	6	42
68239_150613_A_7_3_largeintestine	78	0.57	30	43	35	0.79	4	44	34	0.78	19	51	27	0.71	4	37	41	0.04	7	2	76
57980_145448_A_9_3_largeintestine	36	0.12	6	4	32	0.67	3	15	21	0.45	23	12	24	0.59	2	17	19	0.09	12	0	36
55846_137737_A_9_3_largeintestine	34	0.01	2	0	34	0.69	4	14	20	0.39	25	7	27	0.52	4	11	23	0.28	32	5	29
**Mean Dice**		0.20				0.68				0.44				0.48				0.24			
**Total GT, FP, TP, FN**	3107		533	563	2447		182	1411	1690		1291	1306	1795		390	915	2186		1120	525	2576
**Precision**		0.51				0.89				0.50				0.70				0.32			
**Recall**		0.19				0.46				0.42				0.30				0.17			

Supplementary Figures

Supplementary Figure 1. Number of FTUs in relationship to donor sex, age, ethnicity, BMI, and spatial tissue location. The plots show the number of glomeruli FTUs per square millimeter. Donors are color coded, BMI corresponds to size coding of symbols, squares denote ethnicity with squares indicating White and diamond Black or African American, age is position on x-axis. Slides are numbered by their vertical location in the 3D reference organ, see **Fig. 2**. **a.** Graph for kidney, male. **b.** Graph for kidney, female.

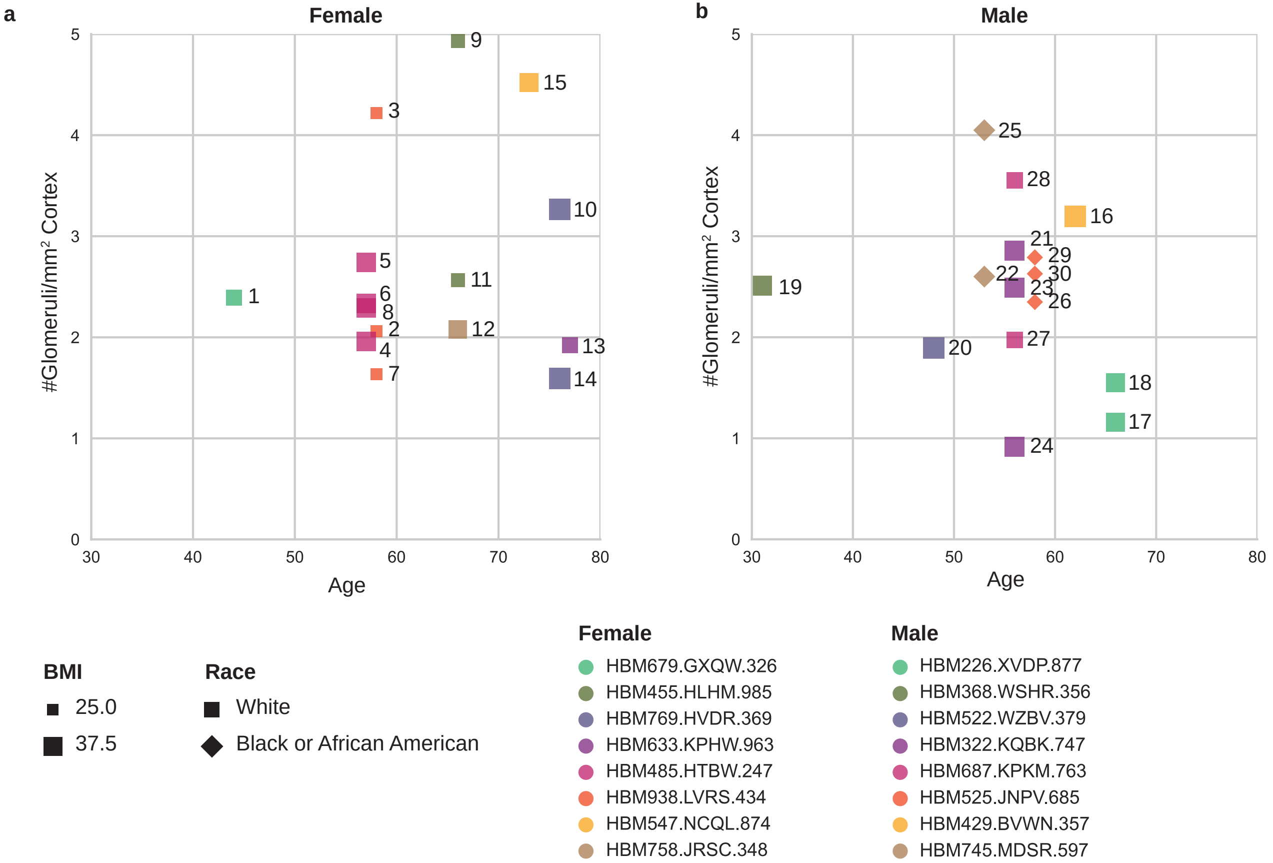
a
Female
#Glomeruli/mm² Cortex
Age
b
Male
#Glomeruli/mm² Cortex
Age
BMI
25.0
37.5
Race
White
Black or African American
Female
HBM679.GXQW.326
HBM455.HLHM.985
HBM769.HVDR.369
HBM633.KPHW.963
HBM485.HTBW.247
HBM938.LVRS.434
HBM547.NCQL.874
HBM758.JRSC.348
Male
HBM226.XVDP.877
HBM368.WSHR.356
HBM522.WZBV.379
HBM322.KQBK.747
HBM687.KPKM.763
HBM525.JNPV.685
HBM429.BVWN.357
HBM745.MDSR.597
